# Completing the picture of field-grown cereal crops: a new method for detailed leaf surface models in wheat

**DOI:** 10.1186/s13007-023-01130-x

**Published:** 2024-02-03

**Authors:** Marie Theiß, Angelina Steier, Uwe Rascher, Mark Müller-Linow

**Affiliations:** Institute of Bio and Geosciences, IBG-2: Plant Sciences, Forschungszentrum Jülich GmbH, Wilhelm-Johnen-Str, 52425 Jülich, Germany

**Keywords:** Leaf angle distribution, Beta distribution, Plant phenotyping, Cereal crops, Stereo imaging, 3D plant reconstruction, Structural leaf model, Leaf angle

## Abstract

**Background:**

The leaf angle distribution (LAD) is an important structural parameter of agricultural crops that influences light interception, radiation fluxes and consequently plant performance. Therefore, LAD and its parametrized form, the Beta distribution, is used in many photosynthesis models. However, in field cultivations, these parameters are difficult to assess and cereal crops in particular pose challenges since their leaves are thin, flexible, and often bent and twisted around their own axis. To our knowledge, there is only a very limited set of methods currently available to calculate LADs of field-grown cereal crops that explicitly takes these special morphological properties into account.

**Results:**

In this study, a new processing pipeline is introduced that allows for the generation of realistic leaf surface models and the analysis of LADs of field-grown cereal crops from 3D point clouds. The data acquisition is based on a convenient stereo imaging setup. The approach was validated with different artificial targets and results on the accuracy of the 3D reconstruction, leaf surface modeling and calculated LAD are given. The mean error of the 3D reconstruction was below 1 mm for an inclination angle range between 0° and 75° and the leaf surface could be quantified with an average accuracy of 90%. The concordance correlation coefficient (CCC) of 99.6% (p-value = $1.5* {10}^{-29}$) indicated a high correlation between the reconstructed inclination angle and the identity line. The LADs for bent leaves were reconstructed with a mean error of 0.21° and a standard deviation of 1.55°. As an additional parameter, the insertion angle was reconstructed for the artificial leaf model with an average error < 5°. Finally, the method was tested with images of field-grown cereal crops and Beta functions were approximated from the calculated LADs. The mean CCC between reconstructed LAD and calculated Beta function was 0.66. According to Cohen, this indicates a high correlation.

**Conclusion:**

This study shows that our image processing pipeline can reconstruct the complex leaf shape of cereal crops from stereo images. The high accuracy of the approach was demonstrated with several validation experiments including artificial leaf targets. The derived leaf models were used to calculate LADs for artificial leaves and naturally grown cereal crops. This helps to better understand the influence of the canopy structure on light absorption and plant performance and allows for a more precise parametrization of photosynthesis models via the derived Beta distributions.

**Supplementary Information:**

The online version contains supplementary material available at 10.1186/s13007-023-01130-x.

## Background

One of the most important phenotypic parameters of a canopy is the angular orientation of leaves [1–4]. It is not only contributing to the complexity of canopy architecture, but also impacting radiation fluxes, photosynthetic capacity and therefore the productivity of the whole plant [5, 6]. The angular orientation of the leaves is described by different parameters, the leaf inclination angle (LIA), the leaf azimuth angle and the leaf angle distribution (LAD) within the canopy [3, 7, 8]. The LIA describes the slope of a leaf with regard to the soil. The LAD refers to the probability to observe a defined inclination angle within a plant canopy. Horizontal leaves in the upper canopy layers intercept most of the incoming light, but they may also cause self-shading and reduce light availability for lower canopy layers. In contrast, erected leaves intercept less light and allow the light to penetrate through the canopy, resulting in a more homogeneous distribution of light in the canopy and an increased carbon gain. Varieties displaying this feature usually have a greater leaf area index and more leaf area exposed to sunlight. For this reason, yield is higher, too [9–11]. For planar-shaped leaves the inclination angle is almost constant. However, leaves of cereal crop plants are very flexible, usually bent along their own axis and the inclination angle varies accordingly [12, 13]. This has a strong impact on the LAD and on the light interception properties of the canopy. As an important factor of various photosynthesis models, the LAD is commonly used there in a parametrized form, the Beta function [14].

Various measurement methods have been developed to determine the angular orientation and LADs of plants, but the characterization of the complex architecture of cereal crops under field conditions holds various challenges, making direct measurements of LAD too time-consuming or indirect determination based on 3D representations very complex, so that there are virtually no validated methods available to date. Their leaves are thin, long, and sometimes twisted around their own axis. Moreover, they are highly flexible and easily moved by wind and thus may change their angular orientation frequently. For this reason, a suitable measurement method should be as insensitive as possible to leaf movements. If the stand becomes denser as it develops, most leaves can only be seen from the nadir position due to the neighboring plants. This limits the ability to obtain a complete 3D representation of individual plants.

Manual measurements, e.g. with an inclinometer [15], are often applied to determine angular orientations of leaves. However, such methods are very time-consuming and consequently, not suitable for larger throughputs in cereal crops because of the complex 3D structure of cereal leaves [16, 17]. Other methods make use of 3D digitizers [18] or laser scanning devices [19] to generate 3D point clouds. These methods are most suited for rigid objects, which do not move during data acquisition. In contrast, photographic methods can be used to collect data for an individual plant within a very short timeframe, typically milliseconds, and therefore they are hardly affected by plant movements. It has been shown in several studies that camera-based methods are suitable to determine plant architecture and leaf angles for agricultural crops [4, 20–26]. A few studies have aimed at addressing the problem of estimating LAD for field-grown wheat plants [19, 24, 27]. Hosoi et al. [19] divided the leaf into segments of 15 mm and calculated the inclination angle for each individual segment. The author stated that the capacity to represent a detailed LAD strongly depends on the segment length alongside the leaf. However, a sufficiently large number of points is needed to have robust surface fittings in each segment. For this reason, it is not possible to used infinitesimal intervals. Dornbusch et al. considered more complex leaf surface models [28, 29]. They reconstructed barley plants from detailed point clouds which were acquired with a 3D digitizer (Digiscan 2000, RSI GmbH, Oberursel, Germany) in the lab. Their approach delivered accurate leaf reconstructions, which considered leaf twisting and bending amongst other factors. However, this methodic approach was not suitable to reconstruct cereal crop leaves under natural conditions. Moreover, their approach was not evaluated with real objects or used to determine leaf-specific parameters like the leaf angle distribution.

With this study, we introduce a processing pipeline for assessing various leaf traits from accurate leaf surface reconstructions of cereal crops with a particular focus on the method evaluation and the usability in field applications. Our approach is based on stereo imaging, which is cheap and easy to implement, nevertheless suitable to deliver detailed leaf models as a basis for reliable analysis of LADs of field-grown cereal crops. An essential step in the modeling is the consideration of the blade curvature by approximating the blade axis with a second-order polynomial as well as the leaf twisting by a leaf twisting function. The computed leaf surface models allow precise and accurate calculation of leaf angles, insertion angles and LADs. Leaf area was included as an additional important surface parameter to judge the quality of the leaf reconstructions. Estimated leaf angles and leaf area were evaluated under outdoor conditions with different experiments. To our knowledge, there are no methods available to collect reference measurements for leaves with a broad LAD. Thus, a more realistic reference model was considered, and the reconstruction quality was evaluated using bent artificial leaves. Finally, the approach was applied to real wheat plants. The calculated LADs were fitted with the Beta function. The comparison of the LAD and the fitted model was used to underline the plausibility of the approach and the applicability of the Beta function estimates for photosynthesis modelling.

## Results

Our approach was validated with two different types of targets, a sphere displaying all possible angles and orientations and artificial leaves with known geometries. The idea behind the sphere experiment was to relate the reconstruction quality to the number of reconstructed points per area (point density ${\rho }_{ss}$) in dependence of the sphere inclination. Method and results of the sphere experiment are explained in the Additional file 9 (Sphere reconstruction) In the following, we focus on the results for the artificial leaf targets.

### Planar leaf model

An artificial flat leaf plant model was arranged with seven different inclination angles. Each inclination angle was imaged four times to evaluate the quality of surface reconstructions. The leaf surface was reconstructed from each pair of stereo images (28 pairs in total). Known leaf parameters (width, area, axis length) were compared to those reconstructed by our data processing workflow (Table 1).

**Table 1:** Reconstructed leaf parameter of the planar leaf model

	Reference	$\overline{x }$	$\sigma$	Percentage change [%]	Variation coefficient
Leaf axis length [mm]	$150$	$155.42$	$6.34$	$+3.6$	$\pm 4.2$
Leaf width [mm]	$11$	$10.74$	$1.32$	$-2.4$	$\pm 1.2$
Leaf area [mm^2^]	$1528$	$1371.23$	$78.54$	$-10.3$	$\pm 5.1$

Reference values and reconstructed values $\overline{x }$, standard deviation $\sigma$, percentage change and variation coefficient: (i) for leaf axis length, (ii) width and (iii) leaf area averaged over all the reconstructed leaves

**Fig. 1:**
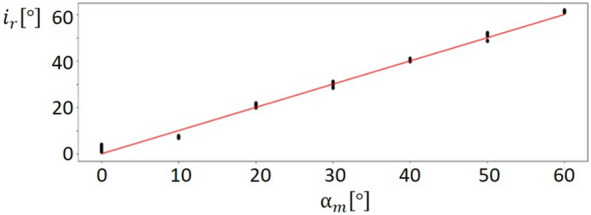
Relation between reconstructed inclination angle ${i}_{r}$ and manually measured inclination angle ${\alpha }_{m}$. The red line marks the identity line. All points are located very close to the identity line (red) indicating a high accuracy of the reconstructed inclination angle

The results for leaf width, leaf axis and leaf area were averaged over all reconstructed leaves. The mean reconstructed leaf width and surface area were slightly smaller than the reference values, while the reconstructed leaf axis length was larger. On average, about 90% of the real surface area was reconstructed. Finally, the reconstructed inclination angle ${i}_{r}$ was compared to the measured insertion angle ${\alpha }_{m}$ (Fig. 1). Most points are evenly and closely distributed around the identity line (red). The correlation between the data points and the identity line was calculated by the concordance correlation coefficient [30] and produced a concordance of 99.6% (p-value = $1.5* {10}^{-29}$). The high correlation to the identity line is also reflected by an accuracy of 0.5° and a precision of 1.72°.

### Bent leaf model

For the next experiment, six different artificial plant models with a bent leaf (150 mm leaf axis length) were analyzed. Parameters like bending radius (${b}_{r}$) and insertion angle (${\alpha }_{Ref}$) are shown in Table 2.

**Table 2:** Leaf bending radius ${r}_{b}$ and insertion angle α for artificial leaf models with bent leaf

Model	${r}_{b}$[mm]	$\alpha$[°]
1	$82$	$35$
2	$155$	$26$
3	$125$	$36$
4	$125$	$44$
5	$155$	$50$
6	$115$	$50$

Each model was imaged four times. Thus, the dataset was composed of 24 stereo image pairs. The leaf area was reconstructed with a comparable accuracy as in the experiment with the planar leaf model. Table 3 summarizes the mean error [%] for the reconstructed insertion angles.

**Table 3:** Reconstructed insertion angle for bent artificial leaf

Model	${\alpha }_{ref}$[°]	${\alpha }_{rec}$[°]	$\mathrm{Mean Error}$[%]
1	$35$	$36.3$	$4$
2	$26$	$26$.2	$0$.7
*3	$36$	$3$ 9.8	$10.5$
4	$44$	$46.7$	$6.1$
5	$50$	$50.2$	$0.4$
*6	$50$	$44.8$	$10.4$

Reference values ${\alpha }_{ref}$ and reconstructed values ${\alpha }_{rec}$, and mean error for insertion angle ${\alpha }.$ *Marks those reconstructions with a mean error above 10%

A high mean error (marked with *) was associated with a high error for one of the image pairs of the model. In the following, a detailed description for model 1 is given (a Table with the reconstructed values for insertion angle, leaf width, leaf area and mean inclination angle and a visual representation of a 3D point cloud and the reconstructed leaf axis and leaf edges are given in the Additional file 1). The reconstructed leaf model represented 97% of the leaf area, which was similar to the results for the planar leaf.

**Fig. 2:**
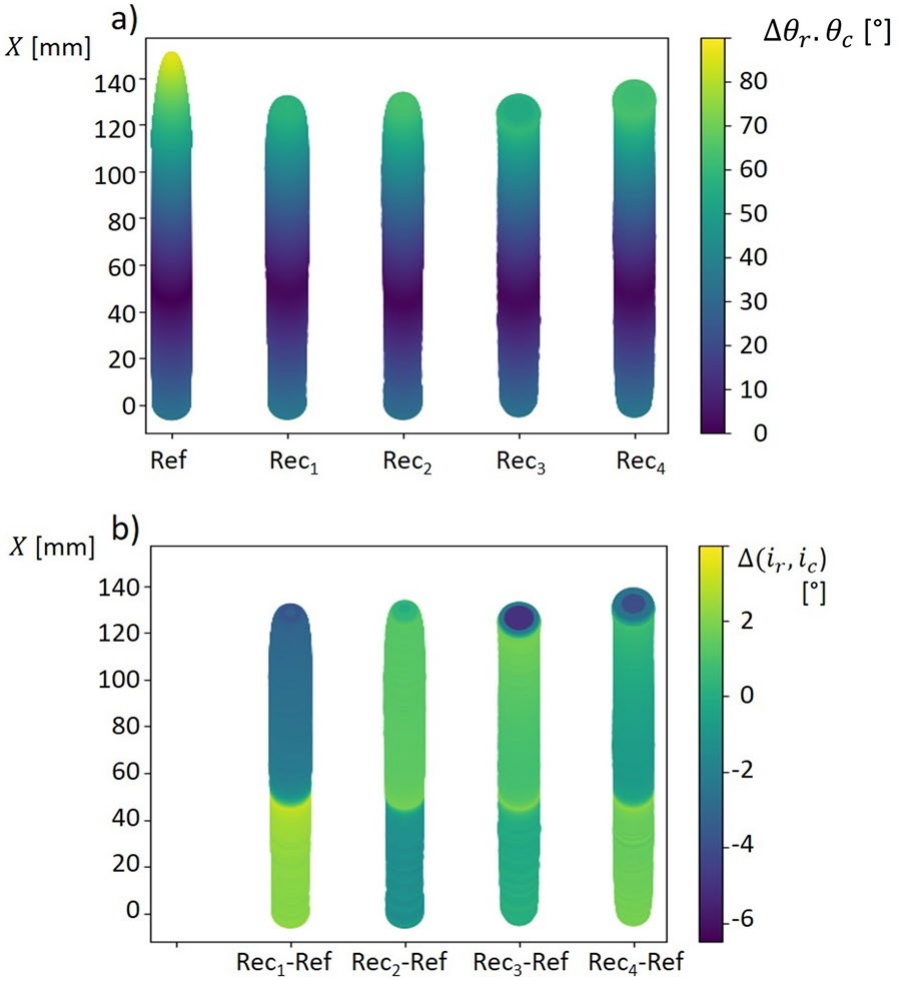
Reconstructed inclination angle along the leaf axis. Plot **a** shows the inclination angle ${i}_{c}$ (Ref) and ${i}_{r}$ (Rec) along the leaf axis $X$. Plot **b** shows the difference between ${i}_{c}$ and ${i}_{r}$. The difference between ${i}_{r}$ and ${i}_{c}$ $\Delta \left({i}_{c},{i}_{r}\right)$ varies between − 6° and 4°. Mean error (ME) and standard deviation σ for $\Delta ({i}_{c},{i}_{r})$ for the four reconstructions (Rec_n_) were: Rec_1_: ME = − 0.43°, σ = 2.38; Rec_2_: ME = 0.37°, σ = 1.22°; Rec_3_: ME = 0.73°, σ = 0.68; Rec_4_: ME = 0.51, σ = 1.05

**Fig. 3:**
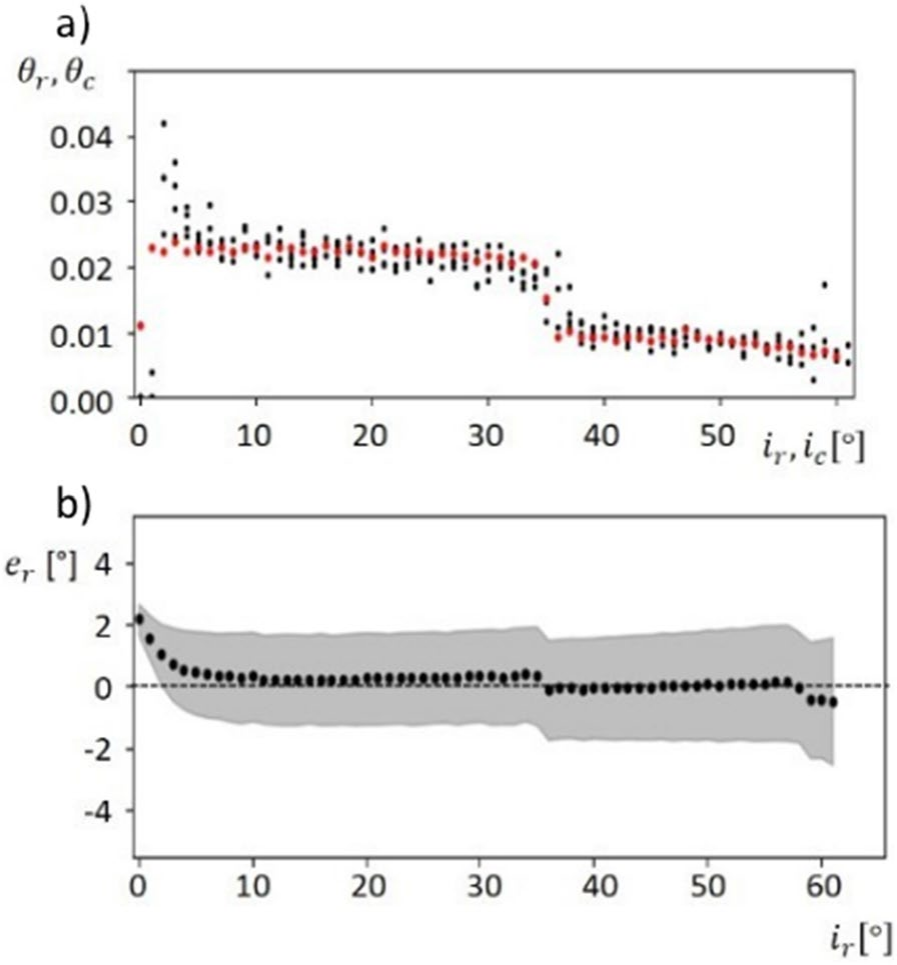
Reconstructed leaf angle distribution and mean error of reconstructed leaf angles. **a** Calculated leaf angle distribution ${\theta }_{c}$ (red) versus reconstructed leaf angle distribution ${\theta }_{r}$ (black). Systematic differences only occurred for inclination angles close to 0°. **b** Mean error (black) and standard deviation (grey area) between the calculated leaf angle distribution ${\theta }_{c}$ and reconstructed distribution ${\theta }_{r}$. The mean error is close to 0° for inclination angles between 10° and 60°. For horizontal surfaces with an inclination angle close to 0°, ${e}_{r}$ shows the highest value of ~ 2°

The reconstructed inclination angle ${i}_{r}$ (which is given by the face normal) was compared to the reference inclination angle ${i}_{c}$. Both angles ${i}_{c}\left(X\right),{i}_{r}\left(X\right)$ follow a similar progression (Fig. 2a)). The insertion angle of 35° is reconstructed with values ranging between 33° and 37.5°. The difference $\Delta ({i}_{c},{i}_{r})$ between ${i}_{r}\left(X\right)$ and ${i}_{c}\left(X\right)$ exhibits an error between − 5.1° and 3.19° (Fig. 2b)). The mean error $\Delta ({i}_{c},{i}_{r})$ for the four reconstructions (Rec_n_) ranges between − 0.43° and 0.73° and σ ranges between 0.68° and 2.38°. At leaf axis position $X\approx 50 mm$, the value of $\Delta \left({i}_{c},{i}_{r}\right)$ shifts from positive to negative and vice versa (Fig. 2b)). The function profile of the reconstructed leaf angle distribution (LAD) ${\theta }_{r}$ is very similar to ${\theta }_{c}$ (Fig. 3a).

All distributions show a predictable drop for ${\theta }_{r}$ and ${\theta }_{c}$ for inclination angles around 35°. Larger deviations were observed for ${i}_{r}<5^\circ$ and ${i}_{r}>50^\circ$. The reconstructed inclination angle ${i}_{r}$ was compared to the reference inclination angle ${i}_{c}$ along the leaf axis for the given leaf blade. The error measure ${e}_{r}\left({i}_{c}\right)$ was used to compare the reconstructed LAD ${\theta }_{r}$ and ${\theta }_{c}$

1 $${e}_{r}\left(X\right)= \left({i}_{c}\left(X\right)-{i}_{r}\left(X\right)\right) (0\le X\le 150)$$

We calculated mean error and σ for each individual inclination angle (Fig. 3b).

Mean error varies between − 0.1° and 0.3° for inclination angles between 10° and 58°. It is higher for inclination angles below 10° and above 58°. The standard deviation varies between 0.5° for ${i}_{r}$ = 0° and 2.05° for ${i}_{r}$ = 61°. In general, higher values in σ were related to higher values for ${i}_{r}$. The highest value for mean error is 2.18° and belongs to an inclination angle ${i}_{c}$ = 0°. Averaged over all inclination angles, the calculated mean error was 0.21° and σ was 1.55°. Analogue Figures for the other models (Additional file 2) and tables with the determined values for insertion angle and mean inclination angle for all models (Additional file 3) are given in the Additional files 2 and 3.

### Field experiment: leaf angle distribution of naturally grown cereal crops

**Fig. 4:**
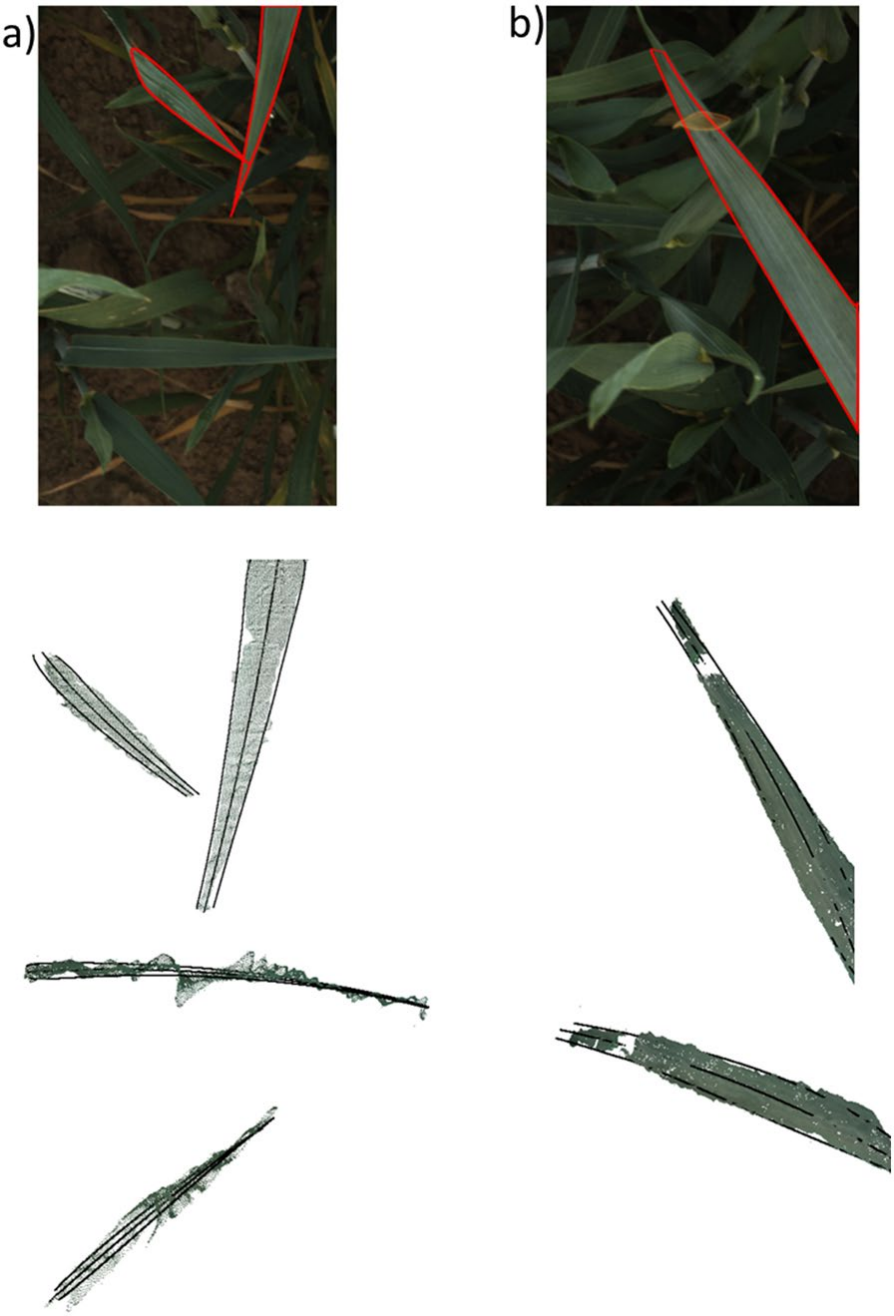
Leaf reconstruction for field grown wheat plants. The stereo image (master camera) shows individual leaves, and the plots show different views of the respective reconstructed 3D point clouds and leaf fits (modeled edges and axis in black)

**Fig. 5:**
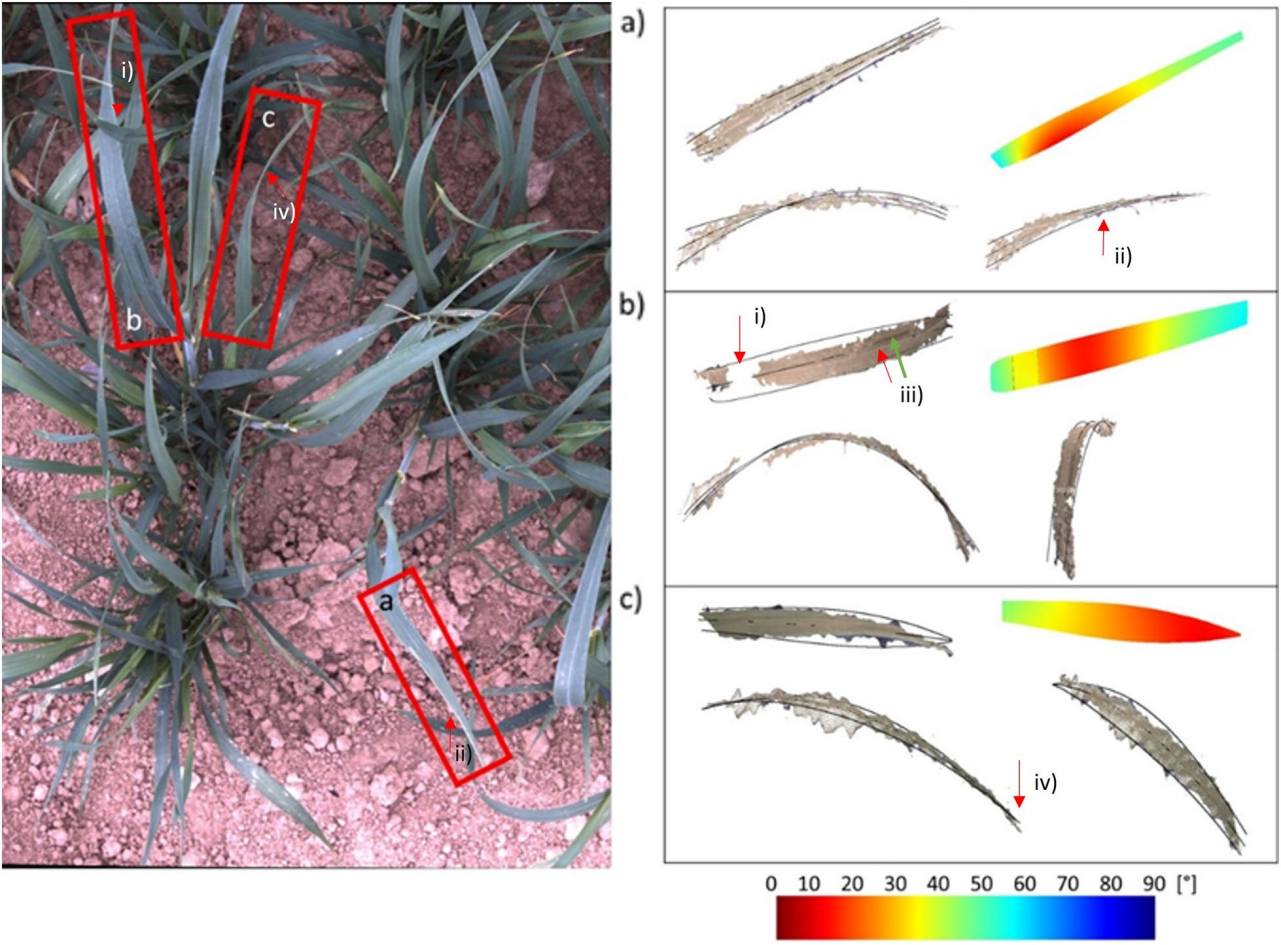
Common errors of leaf reconstructions for field grown wheat plants. The image shows a stereo image (master camera) with three selected leaves. The 3D point clouds show three different views of the respective reconstructed leaf fits (modeled edges and axis in black) and a visualization of the reconstructed leaf inclination angle projected on the leaf model. Leaf a is uniformly twisted around the leaf axis (red arrow iv)). This twisting is visible in the corresponding reconstructions. Leaf b is partly occluded (red arrow i)) by another leaf. For this reason, the 3D-point cloud is fragmented into two parts. However, the leaf model interpolates the missing areas. Moreover, leaf b is tilted laterally due to local twisting and axis bending. Therefore, the reconstructed leaf edges do not fit the border of the 3D-point cloud over the entire leaf length. The leaf axis position in the image (red arrow iii)) differs from the leaf axis fit position (green arrow iii)). Leaf c as a slanted leaf surface. For this reason, the point cloud does not contain the leaf tip

Summer wheat was imaged under field conditions leaves were reconstructed from the images for this case study. In comparison to the utilized artificial targets, leaves of crop plants occlude themselves and they exhibit additional shape features like leaf twisting. Leaf twisting is a common phenomenon for cereal crop leaves, which appears as uniform twisting around the leaf axis. In some cases, twisting and axis bending can combine to an additional 3D surface feature, which looks like a local lateral bending of the leaf axis. Leaf twisting produces additional slanted leaf surfaces, which are sometimes difficult to reconstruct, especially if these surfaces are oriented parallel to the imaging axis. Typical results of the reconstruction and modeling process are exemplarily depicted in Figs. 4 and 5.

**Fig. 6:**
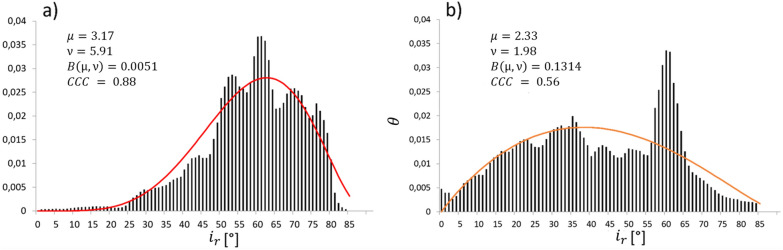
Leaf angle distribution modelled by Beta function. Analysis of two reconstructed field-grown wheat canopies that shows the reconstructed leaf angle distributions *θ* (black bars) and calculated Beta functions with $f\left(t\right)=\frac{1}{B\left(\mu \text{,}\nu \right)}*{\left(1-t\right)}^{\mu -1}*{t}^{\nu -1}$ (**a**) red; (**b**) orange), both displaying a high concordance (CCC > 0.5) between distribution and fit; **a**) CCC = 0.8, and **b**) CCC = 0.56 (Variety: Matthus; sowing density: $250 \frac{seeds}{{m}^{2}})$

To illustrate the influence of the aforementioned factors on particular properties of the leaf surface model, we took a closer look at six sample leaves in Figs. 4 and 6 (red labeled), also to explain the main challenges to retrieve leaf incidence angles in natural canopies. Figure 4 shows three examples, where leaf edges and leaf axis were reconstructed accurately. In Fig. 4b) the leaf is partly occluded by another leaf. Therefore, the 3D point cloud does not contain points in this leaf part, but the leaf model covers this missing area.

i) *Occlusions:* Gaps due to occlusions emerged in leaf b), where the reconstructed 3D-point cloud is fragmented into two parts. Here, the leaf is partly covered by another leaf in the missing area. However, the leaf model could reliably fit these structurally complex leaves and interpolate the missing areas.
ii) *Uniform leaf twisting*: In Fig. 5 right leaf a) is uniformly twisted around the leaf axis, i.e. the leaf tip is inclined towards the right of the image. This twisting is also visible in the corresponding reconstructions (right), were the bottom images clearly show the twisted leaf surface and the reconstructed 3D points, which are located between the fitted leaf edges. The influence of leaf twisting on reconstructed inclination angles is also visible in the top right image. Here, the inclination angle does not change uniformly along the leaf axis, but instead varies between the opposing leaf sides.
iii) *Lateral axis bending*: the leaf axis of leaf b) is tilted laterally due to local twisting and axis bending. This leads to a leaf axis fit, which is not located centrally on the leaf surface. As a consequence, we find overly broad leaf edges, which do not fit the border of the 3D-point cloud over the entire leaf length.
iv) *Slanted leaf surface*: this effect is recognizable for leaf c). Up to the middle it is twisted around the leaf axis followed by a constant tilting in the upper part, which is why the leaf blade is only visible as a thin line from there. The calculated leaf model fits the reconstructed point cloud regarding axis bending and inclination angle. However, the point cloud does not contain the upper part of the leaf and the leaf tip is fitted in the twisting region.

Finally, the LADs and the respective Beta functions of the field-grown wheat plants were computed by processing one stereo image pair per plot with the given pipeline. Assuming that a Beta function fit should reflect the course of the underlying LAD to a certain extent, each distribution and fitted function were compared by computing the Concordance Correlation Coefficient (CCC). Figure 6 shows two examples for a reconstructed LAD (black) and the calculated Beta function (red).

The CCC is not only a measure of the correlation, but also describes the quality of the fitted Beta function with respect to the identity line. In our two examples, the depicted distributions have a CCC = 0.88 and 0.56, respectively (Fig. 6). There are several definitions available, which describe the quality of the CCC. Cohen defined a low correlation for values between 0.1–0.29, a moderate correlation between 0.3–4.9 and a high correlation between 0.5–1.0 [31], while Altman defined the limits like those for the Pearson Coefficient. Values below 0.2 do not indicate any correlation and values above 0.8 describe a high correlation [32]. In our study, the mean CCC was 0.66 and the median 0.68. In 91% of reconstructed leaf angle distributions, the CCC values were greater than 0.5. According to Cohen, this value already indicates a high concordance between the Beta function and the reconstructed LAD. In 22% of cases, the CCC was above 0.8, which Altmann defines as a high correlation, as well.

## Discussion

Measuring leaf inclination angles (LIA) among cereal crops under field conditions is an essential challenge in plant phenotyping. A common practice to collect ground truth data (e.g. for method evaluation or photosynthesis model parametrization) is the usage of inclinometers [19, 33, 34]. However, this approach is time consuming and often difficult in cereal crops because of canopy movements mainly due to wind. The stereo setup is robust against wind-induced canopy movement. However, the setup does not provide a complete 3D reconstruction. Instead, it delivers a 3D point cloud of the canopy surface, which is sensitive to occlusions. Moreover, surfaces with inclination angles > 75° are hardly reconstructed. To overcome this problem the stereo setup could be tilted and images of the canopy could be acquired from different views and angles. Alternatively, a multi view-based camera system could be used [26, 35–37]. These approaches could potentially increase the accuracy and decrease the occlusion-error. However, besides the additional costs, synchronizing and calibrating a multi-setup is far more complicated than a two-camera setup. Photographing from different points of view is time-consuming especially under field conditions and sensitive to canopy movement. For this reason, a multi-view-based approach would be feasible under calm conditions or sing additional enclosures, e. g. in single plant studies. The influence of the light conditions is another important point, which needs to be considered when conducting the acquisition, e. g. by choosing appropriate time points with constant ambient light. Nevertheless, to reduce the impact of light fluctuations, which may occur when clouds are constantly changing, additional artificial illumination could be considered.

In contrast to the available methods, we focused on the application scenarios of our approach, which are primarily but not limited to field research. We consciously decided against a comparison with another, possibly more accurate method and instead validated our approach on a rigid artificial plant model with known geometry leaf properties such as dimensions, area and angular distribution. Therefore, the model was primarily used to validate inaccuracies typically found outdoors like changing light conditions and variations in leaf orientation. In view of this fact, we found very high accuracies in leaf axis length and leaf area (Table 1). To our knowledge, there are no studies, which validate the estimation of leaf surface parameters under outdoor conditions. However, some studies are available, where artificial plant models were used for validation und controlled conditions. Müller-Linow et al. [23] used a wooden plant model with adjustable broad flat leaves, while Dandrifosse et al. [24] used fixed flat crop leaves to determine the accuracy of their approach. However, cereal crop leaves are often bent and twisted along their leaf axes. For this reason, they do not have a constant inclination angle, but each leaf is characterized by an individual leaf angle distribution (LAD). In this study, flat and curved artificial leaf models were used. The evaluation experiment with planar artificial leaves showed high accuracies with a CCC of $99.6 \%$ (p-value = $1.5*{10}^{-29}$). This is in line with the results from previous studies which showed similar accuracies in flat leaves [19, 23, 24]. Since cereal crop leaves are curved along their own axis, a more realistic bent leaf model with a known reference distribution was chosen for the main evaluation experiment. The distribution $\theta$ was reconstructed with a mean error between − 0.43° and 0.73°, which supports the low reconstruction error for flat leaves and punctuates the high accuracy of our reconstruction pipeline. The mean error for inclination angles close to 0° was greater than the mean error for higher inclination angles between 10° and 60°. (Fig. 4). It is known that the reconstruction quality (and the ME) of higher inclination angles can benefit from an error compensation effect [20, 21], while the increase of the ME for lower angles may be attributed to the absence of this effect. The progression of the leaf inclination angle for the bent artificial leaf is determined by two factors, the adjusted insertion angle $\alpha$ and the bending radius ${r}_{b}$. Table 3 shows the mean error for reconstructed insertion angles. High variations were associated with a mismatch of leaf tip and leaf base. This fact did not affect the accuracy for reconstructed LAD. A detailed analysis of the leaf inclination angle was presented for model 1 with $\alpha =35^\circ$ and ${r}_{b}=82 mm.$ The parameters $\alpha$ and ${r}_{b}$ determine the leaf axis position, where the inclination angle is 0°. This position is shifted slightly for the reconstructed inclination angles (Fig. 2b, shift at X ≈ 50 mm). Since the inclination angle is only defined for positive values and $\alpha$ was adjusted to 35°, we expected a jump in ${\theta }_{r}$ at this inclination angle (Fig. 3a). This jump was related to the circular bending and the fact that inclination angles between 0° and 35° occurred twice along the leaf axis.

Recent studies reported photogrammetric methods to estimate plant parameters such as leaf width, leaf axis length and leaf area. However, the proposed methods were not applied to cereal crop leaves [38, 39]. Although leaf area was not the focus of this study, some of the findings will be discussed in the following. Our leaf modeling process underestimates leaf surface area slightly. Overall, 90% of the leaf area for the flat artificial leaf was reconstructed. In comparison, other approaches based on Delaunay triangulation tend to overestimate leaf area by up to 50% due to a stair-step effect [24, 27]. The fact that our pipeline underestimates leaf area results from two factors: Firstly, the fitted leaf model did not cover the given leaf shape perfectly, and secondly, reconstructed leaf edges were sometimes excluded from the fitting process due to edge effects at object corners [40]. This loss of edge points had an impact on leaf width resulting in a narrower leaf model fit. In some cases, more noticeable variations within the point cloud, visible as peaks, were caused by leaves that were close to a reconstructed leaf (Fig. 4a). The points in these areas were not accurately reconstructed and could result in these observed edge effects. However, this case shows the advantage of using a functional description of the leaf surface, which is (within a certain range) robust to such erroneous estimates of the 3D point cloud position. The pipeline calculates a Beta function fit for the reconstructed LAD, which provides a more accurate description of the LAD than one-parametric functions [14]. The average concordance correlation coefficient (CCC) was 0.66, indicating a high correlation between LAD and Beta function [31]. A fit with a very high CCC of 0.88 is shown in Fig. 6a). The shape of the LAD is described very well by the Beta function. Figure 6b) shows the LAD and Beta function with a lower CCC of 0.56. This is a typical example, where the decrease in correlation is mainly caused by a small range in the LAD (here by an overrepresentation of angles between 57° and 65°). This peak was not mapped by the Beta function, while the general distribution of leaf angles is represented by the fit. Wang et al. describe that the Beta function is superior to the ellipsoid function in grasses. This should be evaluated in further analysis [41]. Our modeling approach is applicable to 3D data from various sensory methods like stereo imaging, multi-view setups, LiDAR or structured light imaging, which is employed for example in the commercially available system PlantEye (Phenospex, NL). Depending on scanning time and ambient conditions, LiDAR-based sensors and PlantEye have the potential to provide 3D data with high resolution and accuracy. Nevertheless, to our knowledge there are no studies available that have evaluated the efficiency under outdoor conditions. Maphosa et al. [42] used the PlantEye for wheat phenotyping. The sensor was only applied under controlled conditions and the study did not investigate the properties of the retrieved LADs. Hosoi et al. [19] used a portable scanning LiDAR to acquire high-resolution 3D point clouds. The method evaluation was only demonstrated for still-air conditions. In a typical cultivation scenario, cereal crop plants are moved by wind and long scanning times will likely cause noisy and distorted data. Therefore, the stereo setup applied as single shot approach meets the prerequisites best and is comparatively cheap. The processing pipeline was automated for data from a stereo setup as far as possible to make it applicable to large data sets. However, there are two issues that need to be addressed to improve the processing pipeline. Considering the amount of field data that is usually acquired in experimental studies, one important aspect is to minimize hands-on time during data processing. For this purpose, imaging data should be assigned automatically to field plots via GPS. Moreover, our pipeline still includes time-consuming manual leaf segmentation and segment merging steps, which should be automated when developing the software further. Deep learning-based methods have made noteworthy progress and became more important in plant phenotyping research. These methods could be used to facilitate the opportunity for automatic leaf segmentation [43, 44]. For natural leaves an additional challenge that concerns the leaf edge fitting process was observed. The leaf width function does not include the option to apply different widths for left and right leaf sides, i.e. it requires a leaf axis fit, which is aligned centrally [28]. A more flexible asymmetric leaf edge model could overcome this restriction. The leaf width function is restricted to a long and tapering leaf shape but is not restricted in leaf size. Furthermore, the current workflow focusses on calculating the LAD of leaf blades. However, other organs of cereal crops also contribute to light interception, a topic that should be considered in future studies.

## Conclusions

The leaf angle distribution (LAD) of cereal crops is an important phenotypic parameter as it affects radiation flux and resource efficiency within a canopy. We developed a new evaluated processing pipeline for field applications based on, but not limited to stereo images, which produces detailed models of individual leaves in plant canopies that take the flexible structure of cereal crop leaves into account like leaf bending and twisting. Our evaluation experiment with artificial leaves proved the accurate calculation of LADs ($\theta$). Although LAD is a simplified abstraction of canopy structure, it is an important phenotypic parameter. Therefore, our method will help to get a better understanding of canopy architecture and of the light interception properties. While our leaf surface reconstructions could be used in realistic light interception models, more accurate estimations of LAD would have the potential to increase the quality of photosynthesis models, e.g. by computing and utilizing the Beta function. In addition, the computed leaf models allow for robust estimations of important parameters like leaf area and leaf inclination angle (LIA).

Furthermore, accurate leaf surface reconstructions and better estimations of LAD can be used to study the impact of environmental conditions, climate change and crop management on both canopy structure and leaf morphology. Furthermore, the method can be used for the analysis of leaf morphology. Accurate estimations of LAD also allow to quantify the impact of environmental conditions and crop management on canopy structure. Especially with respect to the effects of climate change resulting e.g. in longer draught periods, studying the adaptation of canopy structure could lead to better insights, how plants are mitigating the effects of sunlight water loss and leaf wilting (e.g. by steeper leaf angles).

A key advantage of the processing pipeline is that is flexible regarding sensors and plants. Thus, it is not restricted to stereo imaging data or wheat plants. It can be adapted for different cereal crops with leaf shapes similar to wheat and it can be applied on for 3D point clouds from various sensors.

## Methods

### Imaging setup

**Fig. 7:**
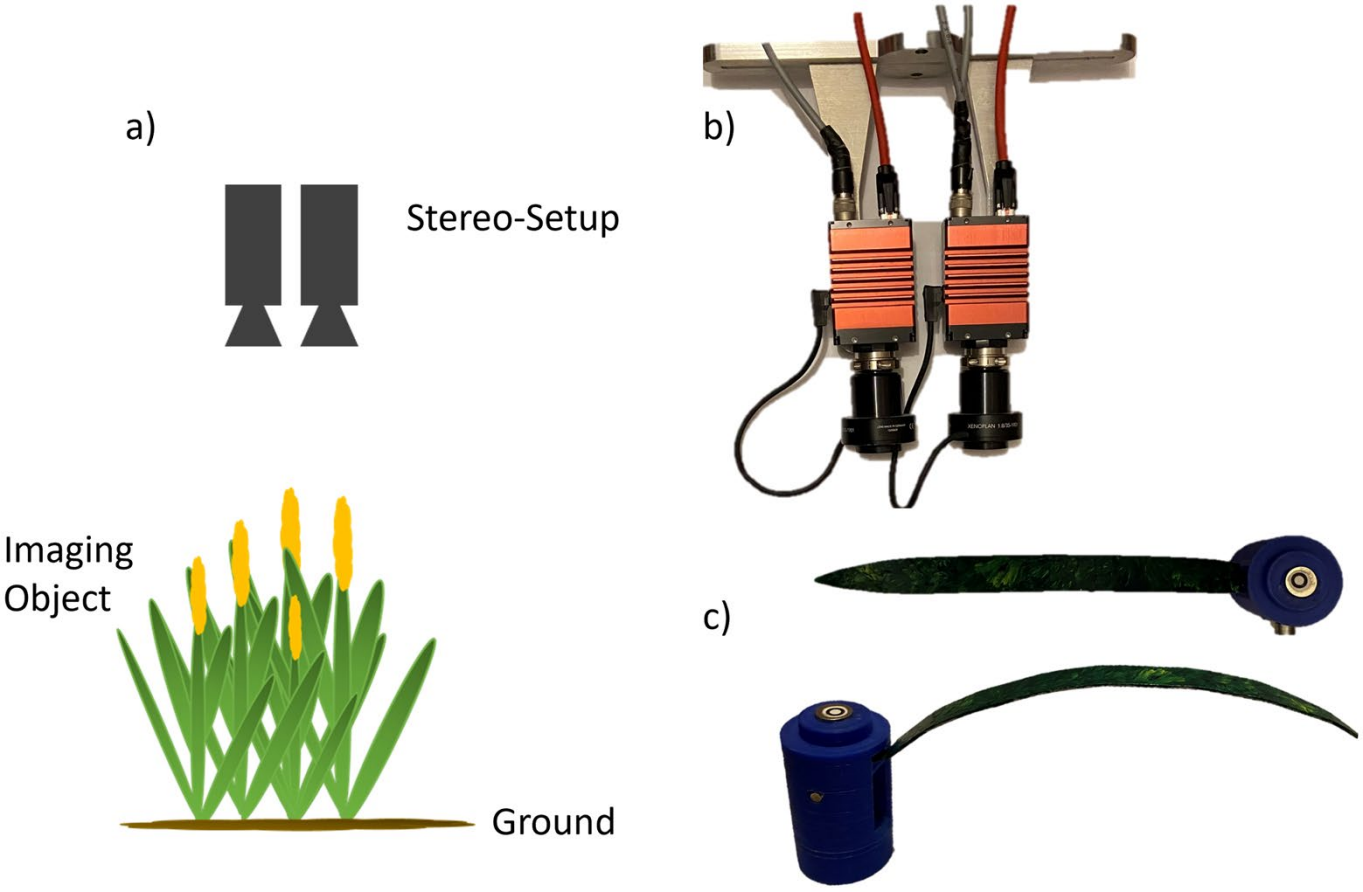
Experimental setup. **a** The stereo-setup was mounted in nadir position to the imaging object. **b** Stereo-setup with two AV Prosilica GT3400C cameras and 35 mm lenses. **c** Artificial plant model composed of a node module and a bent leaf

Two Gig E cameras (*AV Prosilica GT3400C)* equipped with 35 mm lenses *(Schneider Kreuznach APO-XENOPLAN 1,8/35–1901)* were fixed parallel to each other with a baseline of 72 mm. The cameras were inclined to each other, to get a similar field of view. The setup was mounted in a nadir position (90–115 cm) above the target (Fig. 7a))*.*

For simultaneous image acquisition one camera was triggered (master) via software, the second camera (slave) was then triggered via a cable connecting both cameras. The stereo-setup was focused on the upper part of the object. Highlight conditions and shadows were avoided during imaging. All evaluation experiments were carried out outdoors but under obscured conditions. Exposure adaptation to changing light conditions (e.g., to avoid overexposure and bright spots) was controlled via the iso-number, which was set to auto mode. The exposure time was fixed. The stereo camera setup was calibrated with a flat 6 × 7 dot pattern target (22 mm spacing). Camera calibration and image analysis were performed with OpenCV and Python 2.7.6. The stereo camera setup was calibrated with the calibration method of Zhang (here with a maximum number of 1000 iterations and a stop accuracy criterion of 10e−8) [45]. The disparity map was calculated by a modified semi-global block matching algorithm [46, 47] with a block size of one and subsequently transformed to a 3D surface. Instead of the mutual information cost function, the Birchfield-Tomasi sub-pixel metric was implemented [48]. Further analyses were based on rectified stereo images, the obtained disparity map, and the back-projected 3D point cloud. For the validation experiments, we conducted an additional calibration step to correct the orientation of our camera setup in relation to the plant. This ensured that the camera baseline was oriented parallel to the ground. For this purpose, the ground was reconstructed from one of the images and a suitable area for ground calibration was selected manually. A plane was fitted to the ground data points and the plane inclination was used as a correction value for the inclination values in all images.

### Artificial plant model

**Fig. 8:**
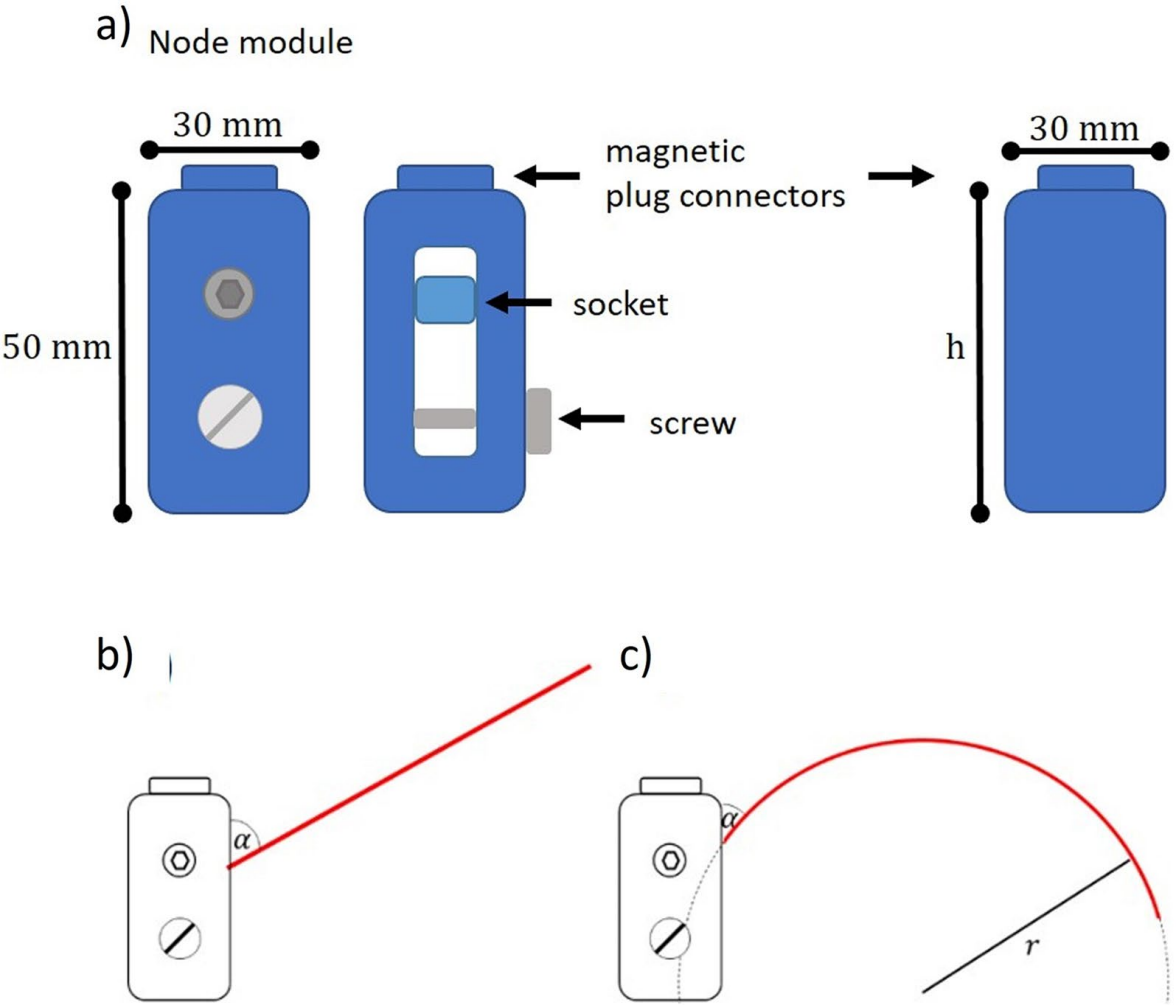
Artificial plant model. **a** The node module fixes the leaf module with a defined inclination angle. The leaf is plugged in a small socket in the module center. The socket inclination is changed by a screw and determines the inclination angle of the fixed leaf (left). The spacer module is used to vary the distance between leaves. They are available in heights (h) of 20 mm, 40 mm and 50 mm (right). Lateral view on **b** planar and **c** bent artificial leaves. The insertion angle α describes the initial inclination angle at the stem. Parameter *r* denotes the leaf bending radius

For method evaluation, a modular artificial plant model (Fig. 7c)) resembling the leaves of cereal crops was designed with *Autodesk® Inventor® 2018 (Build 220,112,000, 112)*. The modular design allowed plants of varying sizes, leaf numbers, leaf inclination angles (angle between a slanted surface and a horizontal plane) and leaf spacing (for plants of different growth stages, e. g. before and after stem elongation) to be assembled. In this manner, it was possible to assemble different plant models with known geometric parameters. The model comprised a leaf module and two types of stem modules (Fig. 8a).

The *leaf module* represented the leaf blade and came in two different sizes. Leaf modules were laser-cut from thin (1 mm) aluminum sheets. Their shape resembled wheat leaves. We decided to build the leaves from bendable thin metal plates, which offered a high flexibility to create various leaf geometries. The leaves’ cross-sections needed to be very thin. According to manufacturer informationleaves of the required length would break easily. The leaf surface was colored with acrylic paint to produce irregular textures with various tones of green. An irregular pattern was chosen to facilitate the stereo reconstruction process. At the same time, our aim was to create a surface that has comparable reflective properties to natural leaves. The thin material maintained its shape and allowed leaf bending to be adjusted by hand. The leaf blade had a leaf axis length of 150 mm, a maximal width of 11 mm and a leaf area of 1528 mm.

Both types of stem modules (node module and stem module, Fig. 8a) were colored with a blue paint and produced with a 3D printer (*Felix Pro2, FELIXprinters, Technology FDM, plane-thickness 0.05 mm, Material PLA*). The blue color gives the advantage that leaves can easily be segmented from stem modules during image processing. The modules have a cylindrical shape of 30 mm in diameter with magnetic plug connectors at the top and bottom.

### Image processing

For stereo 3D reconstruction, the semi-global block matching algorithm was applied as described in the imaging setup. Further processing steps were applied to the resulting disparities in order to create an image with labeled areas that correspond to the wheat leaves or the evaluation object (sphere, artificial leaf). Pixels with unknown disparity and those containing background were masked out. Background pixels were identified via color channel thresholding in HSV-space [49]. The filtered disparity map then contained areas that mainly represented leaves or leaf parts. A connected components algorithm implemented in OpenCV [50] was applied to search for connected pixels in the disparity map and labeled related areas. We cannot rule out the possibility that the disparity map still contained background pixels. For this reason, a manual editing step was added to either (i) cut components that contained different leaves (which may occur e. g. in overlapping leaves), (ii) remove components that did not represent leaf parts, or (iii) join components that belong together, but were not reconstructed as a whole. The perspective transform matrix, obtained by stereo calibration, was used to project these disparities back into 3D space. In the following step, leaf angle distributions (LAD) were calculated from our 3D point clouds. Therefore, they were further processed to calculate a surface mesh, which is built up from triangles called faces. Each face is represented by an inclination angle and a face area. The sum of all face areas represents the leaf area. Since both parameters, surface area and inclination angle, affect LAD, both variables were evaluated independently.

### Further processing and experiment evaluation

The preliminary experiment with the sphere target is described in detail in the Additional file 9 (Sphere reconstruction). In the following, the method evaluation with different types of artificial leaf models is described. A visualization of an artificial model is shown in Fig. 7c) and Fig. 8. In the Evaluation Experiment Planar Leaf Model (Fig. 8b)), the potential to reconstruct surfaces shaped like planar cereal crop leaves was determined. Estimated parameters were leaf width and leaf area for a planar artificial leaf with different insertion angles α (see Fig. 8), which is the initial inclination angle between the stem and node. The focus of this experiment was the quality of a surface, reconstructed from 3D point clouds. The assumption was that the number of points reconstructed for a given area affects the resolution of a fitted surface. In the Evaluation Experiment Bent Leaf Model (Fig. 8c)), we combined the previous questions and determined the accuracy of leaf angle reconstruction. An artificial plant with a curved leaf was used for this experiment and reconstructed LAD were compared to calculated reference results. Realistic field data were processed to calculate LAD of cereal crops and a Beta function was used to model the distribution.

### Planar leaf model

For this experiment, an artificial plant that contained a small flat leaf blade (Fig. 8b) was assembled to compute the accuracy of leaf angle, leaf width and leaf area measurements, respectively. For imaging, the aperture was set to a value to get the whole object in focus. Although the leaves of our artificial model did not move, camera exposure was adjusted according to the field application, meaning that short exposure times were used to avoid blurred regions that may occur due to leaf movement. To figure out an optimal exposure value, a gray panel calibration was used. For this purpose, a gray panel was imaged several times, thereby decreasing the exposure time continuously until the gray value remained unchanged in the image. A dataset with 28 image pairs was prepared, including images with different leaf inclination angles and leaf orientations. The initial leaf angle, the insertion angle $\alpha$ (the inclination angle at the node), was adjusted between 0 and 60° in steps of 10°. For each insertion angle α, four images with different plant orientation (model rotation around its own axis in steps of 90°) were taken. A protractor *(Leitz)* was used to measure ${\alpha }_{m}$ manually.

### Bent leaf model

For this experiment, a small leaf was bent in the shape of a circle with a defined radius and assembled with a node module with insertion angle α (see Fig. 8c). Each of the resulting six different models was imaged four times. The stereo cameras were set up in the same way as in the planar leaf experiment. Angle $\alpha$ was fixed and manually measured with a protractor *(Leitz).* An overview of all models (bending radii ${r}_{b}$ and insertion angle $\alpha$) in this experiment is given in Table 2. To evaluate our processing pipeline, the known geometry of our artificial leaf was used to get reference values for the LAD and leaf inclination angle (LIA) along the leaf axis. We calculated 1500 evenly distributed values for LIA ${i}_{c}\left(X\right)$ and the LAD ${\theta }_{c}$ from the leaf width, leaf length, insertion angle, and radius of circular curvature. Parameter $X$ denotes the position along the leaf axis in the range of 0 mm (at the leaf node) and 150 mm (at the tip). LIA ${i}_{c}\left(X\right)$ was determined in steps of $\Delta X=0.1{\text{mm}}$ and approximated the leaf area for the related interval ${a}_{[X,\Delta X]}$.

For computation of ${\theta }_{c}$, angles between 0° and 90° were considered and inclination angles were computed along the leaf axis by:

2 $${i}_{c}\left(X\right)= \left|\left(2+acrsin\frac{X}{2*{r}_{b}}\right)-\alpha \right| \, \mathrm{with } \, (0\le X\le 150)$$

The total leaf area $A$ is then given by:

3 $$A=\sum_{{i}_{cal}=0}^{90}{a}_{{i}_{cal}}$$

Subsequently, the LAD was computed from the total leaf area and the leaf area with a given inclination angle as follows:

4 $${\theta }_{c}=\frac{{a}_{{i}_{c}}}{A} \,\, (0\le {i}_{c}\le 90)$$

### Leaf modeling process

**Fig. 9:**
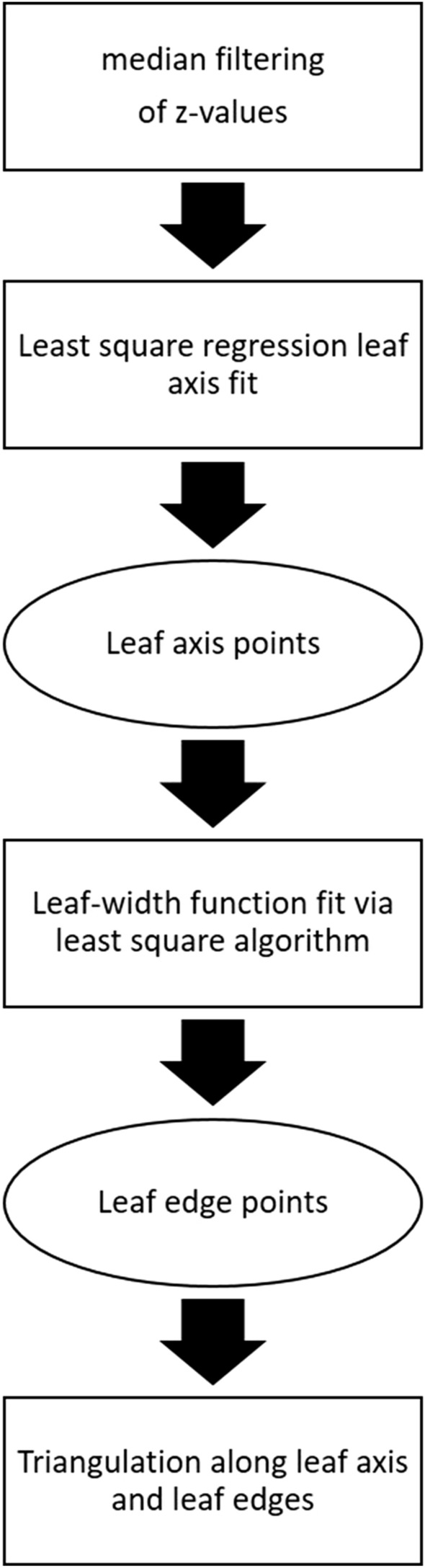
Flow chart of the leaf modeling process. Before leaf fitting, a cluster-wise median filtering of $\mathrm{p{\prime}}$ was applied

The 3D data was computed from stereo images as described in the Image processing paragraph. The 3D data points $p$ passed through different processing steps to generate leaf surface models and finally, to calculate the leaf inclination angle and LAD. The processing pipeline is depicted in Fig. 9.

**Fig. 10:**
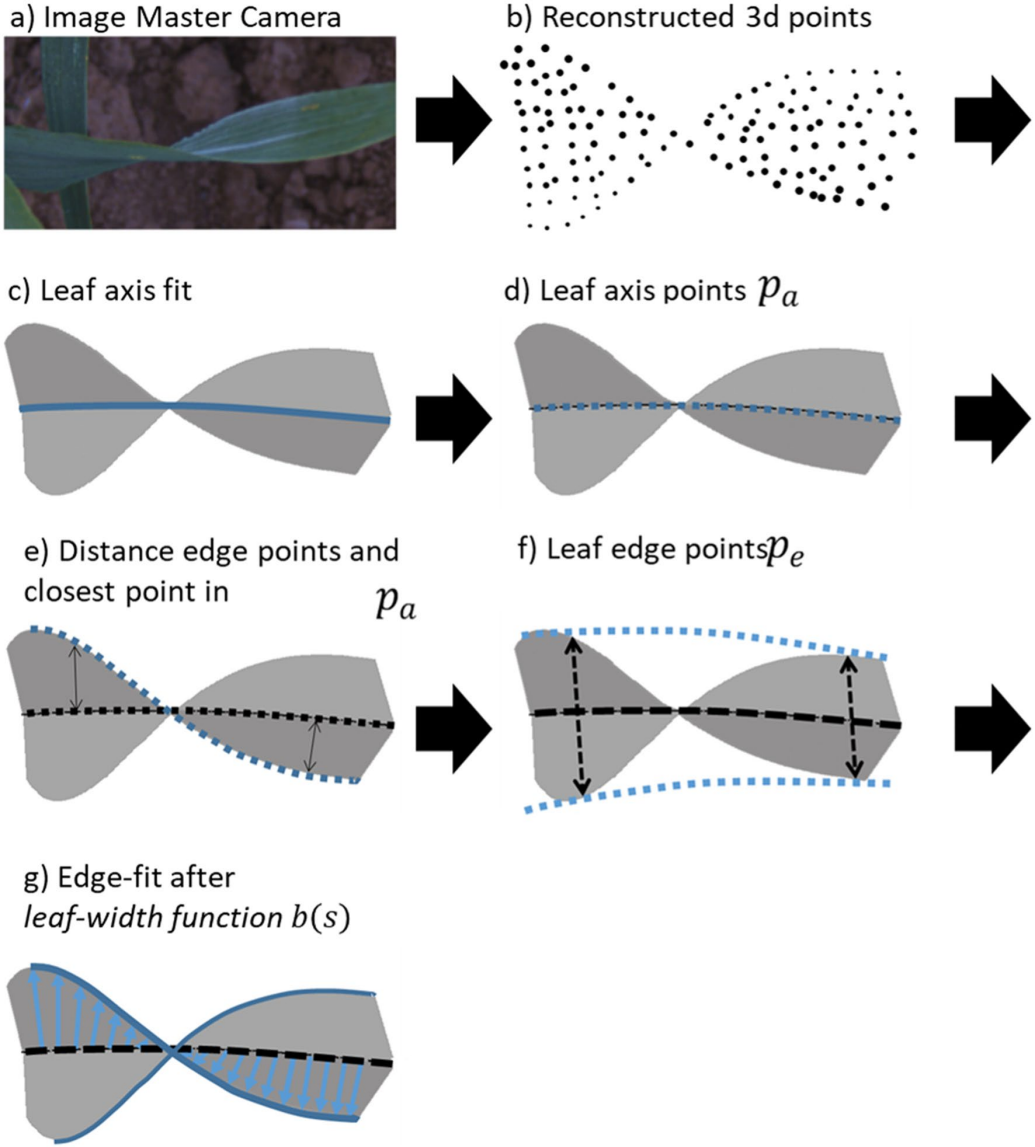
Detailed visualization of the leaf fitting process from points cloud to mathematical leaf model

The point cloud of each individual leaf was smoothed with respect to the z-value to reduce noise caused by pixels or 3D points that do not belong to the leaf (Fig. 10b)). Therefore, 3D points were projected onto the x–y plane and meshed via Delaunay triangulation [51] to identify the neighbors of each pixel, i.e. pixels that are directly connected. Afterwards, the z-value of this pixel was corrected by the *median z-value* of all connected neighbors (this pixel included). The surface fitting process is based on the identification of the leaf axis and leaf edges. In contrast to Dornbusch et al. [28, 29], we also implemented a fitting approach for the estimation of the leaf axis position. For the two different leaf shapes, separate fitting functions were computed via *least squares regression*, one which represents the leaf axis in the flat leaf and one for the bent leaf. In the data points $p{\prime}$ of the flat leaf a line was fitted and in the bent leaf a circle function was fitted. The resulting fitting functions (Fig. 10c) were used to calculate a new set of points ${p}_{a}$ (Fig. 10d) located along the *leaf axis*. $p$ was split up into two groups associated with either the right or left side of the leaf blade with respect to the leaf axis. The Euclidean distance was determined to the closest axis points ${p}_{a}$ (Fig. 10e). Afterwards two subsets ${p}_{e}$ were calculated which represented the opposite leaf edges (Fig. 10f). In the next step, a leaf edge fit was determined by the *leaf-width function* $b\left(s\right)$ (Fig. 10g). Therefore, the relative axis positions ${s}_{i}$ of the axis points ${p}_{a}$ were calculated according to [12, 28, 29].

5 $${s}_{i}=\frac{i-1}{{n}_{{P}_{a}}-1}\left(1 \le i \le {n}_{{P}_{a}}\right)$$

$b\left(s\right)$ is then computed with:

6 $$b\left(s\right)={b}_{max}*\frac{{c}_{2}*\left({c}_{1}+s\right)*{\left(1-s\right)}^{{c}_{2}}}{{\left[\left(1+{c}_{1}\right)*\left(\frac{{c}_{2}}{{c}_{2}+1}\right)\right]}^{{c}_{2}+1} } \,\, (0\le s\le 1;{b}_{max},{c}_{1},{c}_{2}\ge 0;{c}_{1}*{c}_{2}\le 1)$$

A least square trust region reflective algorithm implemented in the scientific computing Python library scipy [52] was used to optimize the parameter ${b}_{max}$ (maximum leaf width) as well as the leaf curvature ${c}_{1}, {c}_{2}$. We used ${c}_{1},{c}_{2}, {b}_{max}$ and ${s}_{i}$ to calculate a set of points along the *leaf edges* ${p}_{e}$. The final fit was represented by two point sets, namely ${p}_{a}$ and ${p}_{e}$ (shown in Additional file 4). Fitted leaf points were used to triangulate leaf edges along the leaf axis and to calculate the leaf area $a$ for each face and the corresponding inclination angle ${i}_{r}$ from each face normal. This mesh represented the final *leaf model.*

### Field validation: quantification of leaf inclination in summer wheat

Summer wheat *Triticum aestivum L.* Matthus was sown on 27 March 2020 in 3 × 3 m plots with a density of 150, 250, 350 and 450 seeds/m^2^ at Campus Klein-Altendorf, Germany (50° 37′ 29″ N and 6° 59′ 06″ E). The experimental design is shown Additional file 5. The plots were treated with nitrogen $80\frac{kg N}{ha}$ (04/17/2020) and sprayed with herbicide (04/30/2020) and fungicide (05/14/2020). The stereo imaging setup was mounted on a hand-driven field platform “Field4Cycle” [53] and the distance between the ground and the imaging setup was fixed at 1.35 m resulting in a field of view of about 40 cm × 30 cm (Additional file 6). Images were collected on 25 May 2020 between 10 and 11 am. The imaged plot segment was shaded to avoid direct sunlight and used the gray panel calibration (see *Methods, Planar Leaf Model*) to adjust exposure time. Images were processed as described in the *Image processing* section. Cereal crop leaves are often twisted slightly along their own axis. This property was described by Dornbusch et al. [29]. For this reason, the leaf modeling process was extended by an additional fitting step, which models the leaf twisting. The surface-twist function $\psi (s)$ quantifies leaf twisting along the leaf axis:

7 $$\psi \left(s\right)={\psi }_{0}+\Delta \psi *s\frac{\left(1+{c}_{3}*s\right)}{1+{c}_{3}}.$$

It is defined by the relative axis position of leaf axis point $s$, the rotation angle ${\psi }_{0}$ at the node and the difference between the distal angle and the rotation angle at the node $\Delta \psi$ [28]. The parameter ${c}_{3}$ describes the curvature of leaf twisting. Optimal values for the functional parameters of $\psi (s)$ were calculated by minimizing the distance between the reconstructed 3D points and the fitted surface points via the least squares method. While the position of the leaf axis ${p}_{a}$ remains unchanged, the position of the leaf edges ${p}_{e}$ is changed by $\psi (s)$. The final leaf fit is meshed as described in the *Leaf modeling process* section. Based on the leaf model the LAD was reconstructed. A common way to describe the LAD in a parameterized way is the Beta function, which was fit into the calculated LAD [14].

### Supplementary Information

**Additional file 1.** Reconstruction results of bended leaf. Reconstructed values for insertion angle, leaf width, leaf area and mean inclination angle for bent leaf model 1 are given in the table. The plot figure below shows the reconstructed 3D point cloud (green) and the fitted leaf model (leaf axis and leaf edges in black).

**Additional file 2.** Reconstructed inclination angle along the leaf axis, leaf angle distribution and mean Error of reconstructed leaf angles. Plot a) Model 2, b) Model 3, c) Model 4, d) Model 5 and e) Model 6: show the inclination angle ${i}_{c}$ (Ref) and ${i}_{r}$ (Rec) along the leaf axis $X$ (top). The mean error (black) and standard deviation (grey area) between the calculated leaf angle distribution ${\theta }_{c}$ and reconstructed distribution ${\theta }_{r}$ (middle). The Calculated leaf angle distribution ${\theta }_{c}$ (red) versus reconstructed leaf angle distribution ${\theta }_{r}$ (black) (bottom).

**Additional file 3.** Reconstructed values for insertion angle, and mean inclination angle for bent leaf model 2–6.

**Additional file 4.** Illustration of the leaf fitting method. Red dots are the initial points $p\left(x,y,z\right)$. Black line consists of individual dots, representing leaf axis points ${p}_{a}\left(x,y,z\right)$ and leaf edges ${p}_{e}\left(x,y,z\right)$.

**Additional file 5.** Color-coded plot design for field experiment. Three different varieties were sown with four sowing densities. The left Table shows the corresponding varieties and sowing densities.

**Additional file 6.** Stereo imaging setup mounted on the hand-driven “Field4Cycle”.

**Additional file 7.** Point cloud density ${\rho }_{ss}$ for the projected area. The bar chart shows the point cloud density ${\rho }_{ss}$ for the projected (visual) area of different spherical segments. Segments were defined by ${i}_{r}$, segments ranged between ${t}_{L}$-${t}_{H}$. 3D points were reconstructed for all spherical segments. It is apparent that the number of points per projected area decreases for surfaces with an inclination angle above 60°. The boxplots in the upper part of the figure depicts the reconstruction error $d(p,s)$ for all points grouped by the inclination angle. A stronger deviation of $d(p,s)$ values indicates less precision in the 3D reconstruction, while a mean value for $d(p,s)$ (red dotted line) close to 0 is an indicator for high reconstruction accuracy.

**Additional file 8.** Process to approximate the real sphere center. A sphere ${s}_{g}$ is fitted to the data points $p$. The sphere is defined by ${c}_{g}$ and ${r}_{g}$. Following this ${c}_{g}$ is used as a starting point to fit a sphere ${s}_{f}$ with the real radius ${r}_{m}$ to the p.

**Additional file 9. **Sphere reconstruction.

## Data Availability

The datasets used and/or analyzed during the current study are available from the corresponding author on reasonable request.

## Abbreviations

*A*: Total leaf area
*a*_*i*_: Leaf area with a given inclination angle
*a*_*i*_, *α*_*m*_: Insertion angle
*b*(*s*): Leaf width function
*b*_*max*_: Maximum leaf width
*c*_1_, c_2_: Leaf curvature parameter
*c*_3_: Curvature parameter of leaf twisting
*c*_*f*_: Sphere center position in reconstructed 3D space *c*_*f*_ (*x*, *y*, *z*)
*c*_*g*_: Initial sphere center position *c*_*g*_ (*x*, *y*, *z*)
*d*(*p*, *s*): Euclidian distance between $p$ and ${s}_{f}$
*e*_*r*_(*i*_*c*_): Difference between ${i}_{c}(X)$ and ${i}_{r}(X)$
*i*_*c*_(*X*): Calculated inclination angle along the leaf axis
*i*_*r*_, *i*_*c*_: Reconstructed, calculated inclination angle
LAD: Leaf angle distribution
ME: Mean error. Accuracy
*nP*_*a*_: Number of leaf axis points
*n*_*ss*_: Number of points in a spherical segment
*O*_*ss*_: Visual area of a spherical segment
*θ*_*c*_, *θ*_*r*_: Leaf angle distribution, calculated and reconstructed
*p*ʹ: Point cloud
*p*: Point in reconstructed 3D space *p*(*x*, *y*, *z*)
*p*_*a*_: Leaf axis points *p*_*a*_(*x*, *y*, *z*)
*p*_*e*_: Leaf edge points *p*_*e*_(*x*, *y*, *z*)
*p*_*ss*_: Spherical segment
*ρ*_*ss*_: Point cloud density of a spherical segment
*r*_*b*_: Bending radius artificial leaf
Rec_1_/Rec_2_/Rec_3_/Rec_4_: Different leaf reconstructions
Ref: Reference / calculated leaf reconstruction
*r*_*g*_: Initial sphere radius
*r*_*m*_: Hand measured sphere radius
*S*: Leaf axis length
*s*_*i*_: Relative axis position
*s*_*f*_: Final sphere fit *c*_*f*_ (*x*, *y*, *z*)
*s*_*g*_: Initial sphere fit *c*_*g*_ (*x*, *y*, *z*)
σ: Standard deviation, precision
*t*_*L*_*, t*_*H*_: Inclination angle of lower and upper boundaries of spherical segments
*X*: Leaf axis
$\psi (s)$: Surface-twist function
${\psi }_{0}$: Axial rotation angle
$\Delta \psi$: Difference between basal and axial rotation angle
