# Supplementary figures and images for: Completing the picture of field-grown cereal crops: a new method for detailed leaf surface models in wheat
Source: Plant Methods. 2024 Feb 3;20:21. doi: 10.1186/s13007-023-01130-x (PMC10837940; doi:10.1186/s13007-023-01130-x)

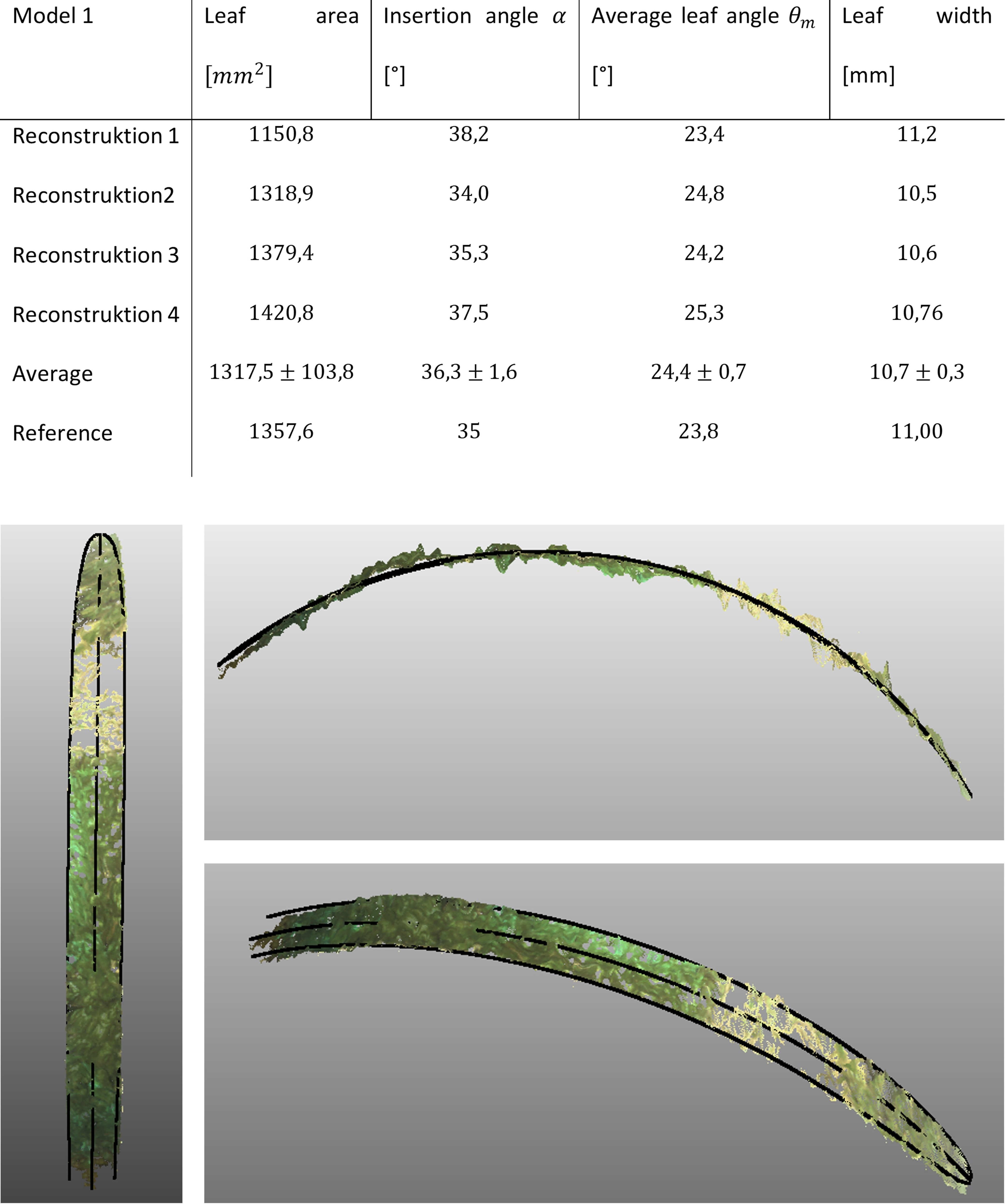

Supplement: Supplementary file 1 — Additional file 1. Reconstruction results of bended leaf. Reconstructed values for insertion angle, leaf width, leaf area and mean inclination angle for bent leaf model 1 are given in the table. The plot figure below shows the reconstructed 3D point cloud (green) and the fitted leaf model (leaf axis and leaf edges in black). [file 13007_2023_1130_MOESM1_ESM.jpg]

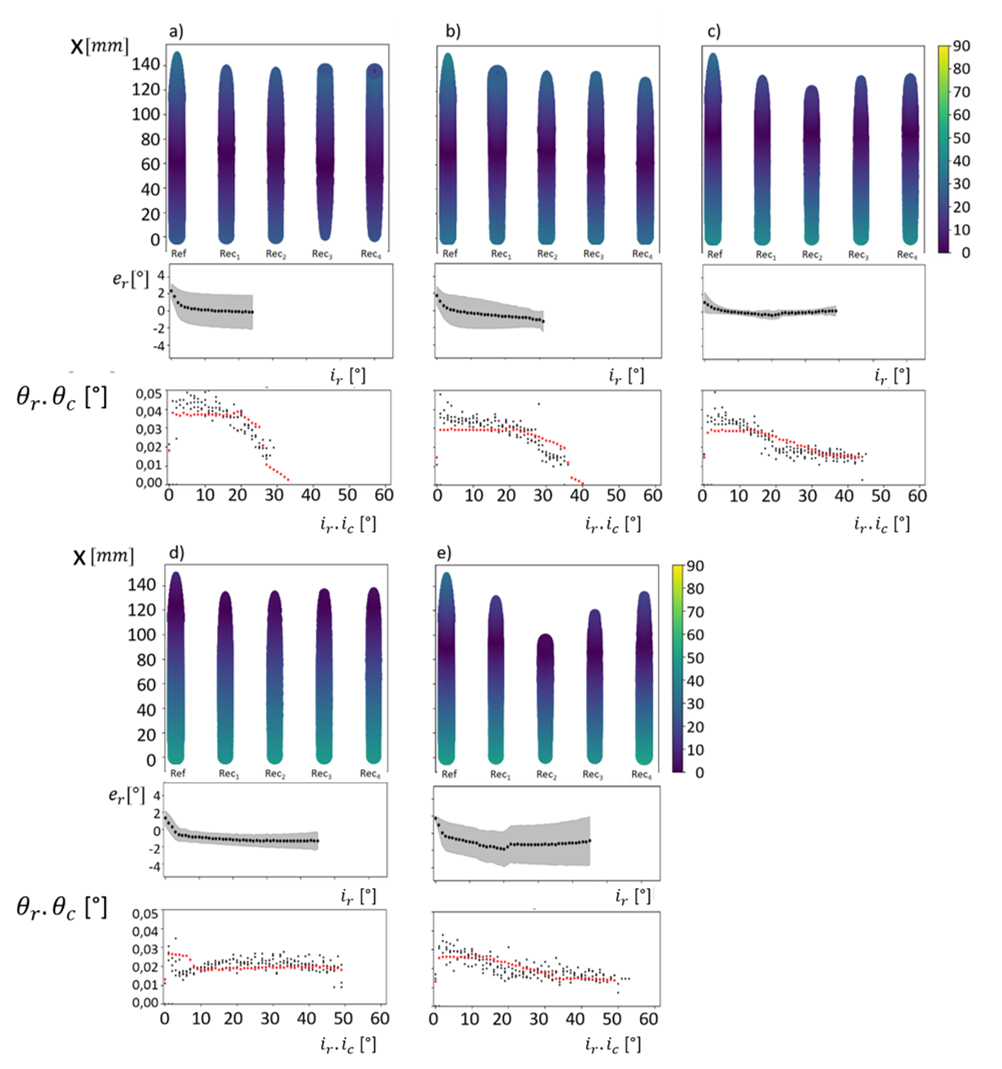

Supplement: Supplementary file 2 — Additional file 2. Reconstructed inclination angle along the leaf axis, leaf angle distribution and mean Error of reconstructed leaf angles. Plot a) Model 2, b) Model 3, c) Model 4, d) Model 5 and e) Model 6: show the inclination angle ${i}_{c}$ic (Ref) and ${i}_{r}$ir (Rec) along the leaf axis $X$X (top). The mean error (black) and standard deviation (grey area) between the calculated leaf angle distribution ${\theta }_{c}$θc and reconstructed distribution ${\theta }_{r}$θr (middle). The Calculated leaf angle distribution ${\theta }_{c}$ [file 13007_2023_1130_MOESM2_ESM.jpg]

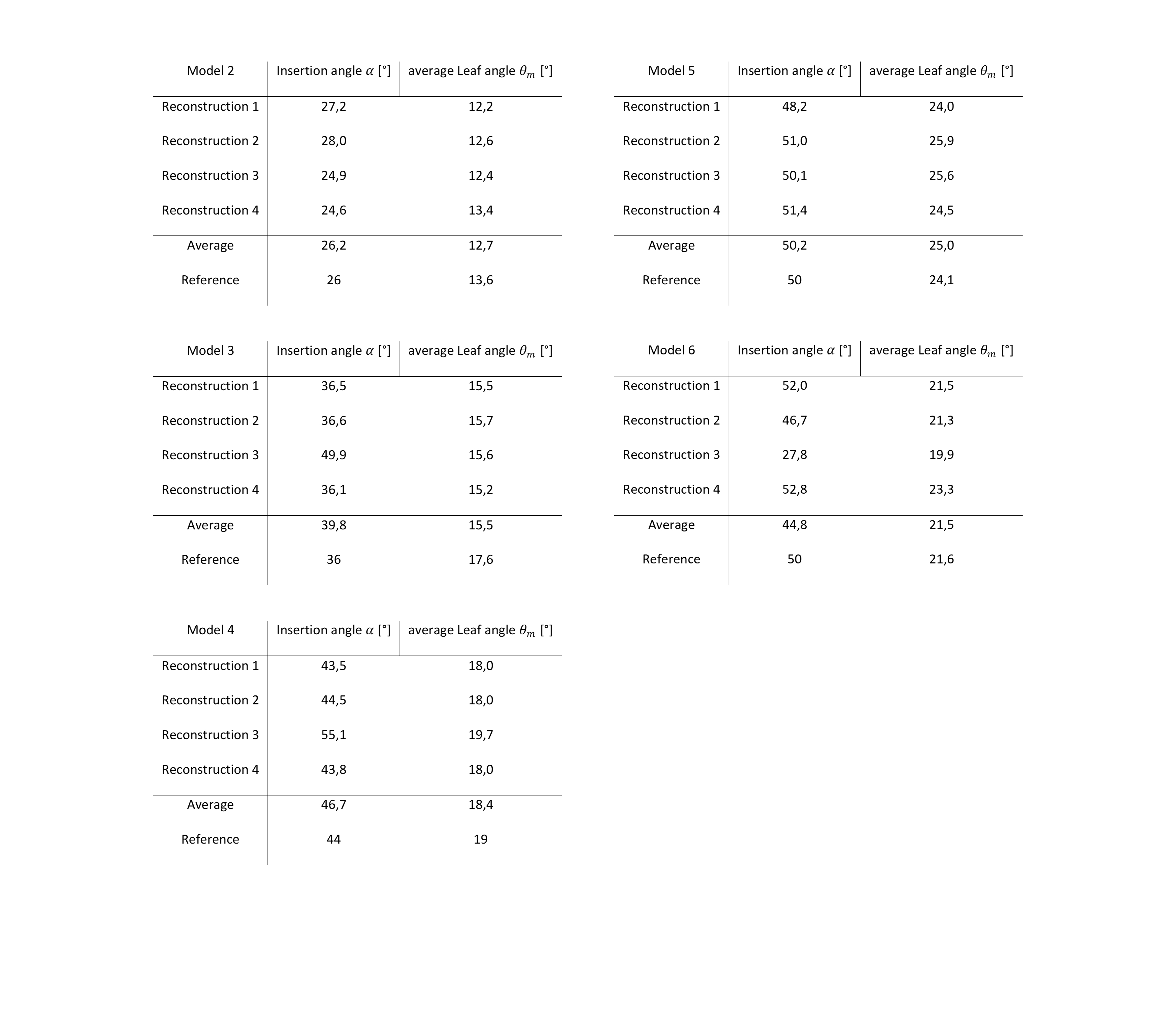

Supplement: Supplementary file 3 — Additional file 3. Reconstructed values for insertion angle, and mean inclination angle for bent leaf model 2–6. [file 13007_2023_1130_MOESM3_ESM.jpg]

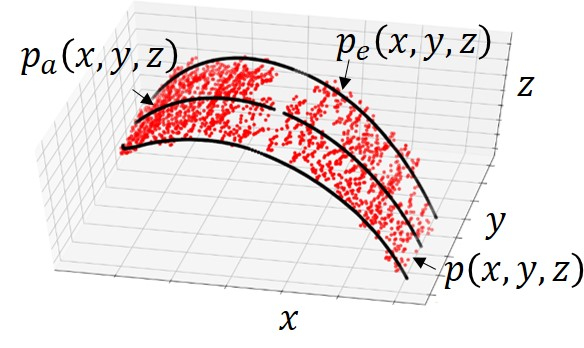

Supplement: Supplementary file 4 — Additional file 4. Illustration of the leaf fitting method. Red dots are the initial points $p\left(x,y,z\right)$px,y,z. Black line consists of individual dots, representing leaf axis points ${p}_{a}\left(x,y,z\right)$pax,y,z and leaf edges ${p}_{e}\left(x,y,z\right)$pex,y,z. [file 13007_2023_1130_MOESM4_ESM.jpg]

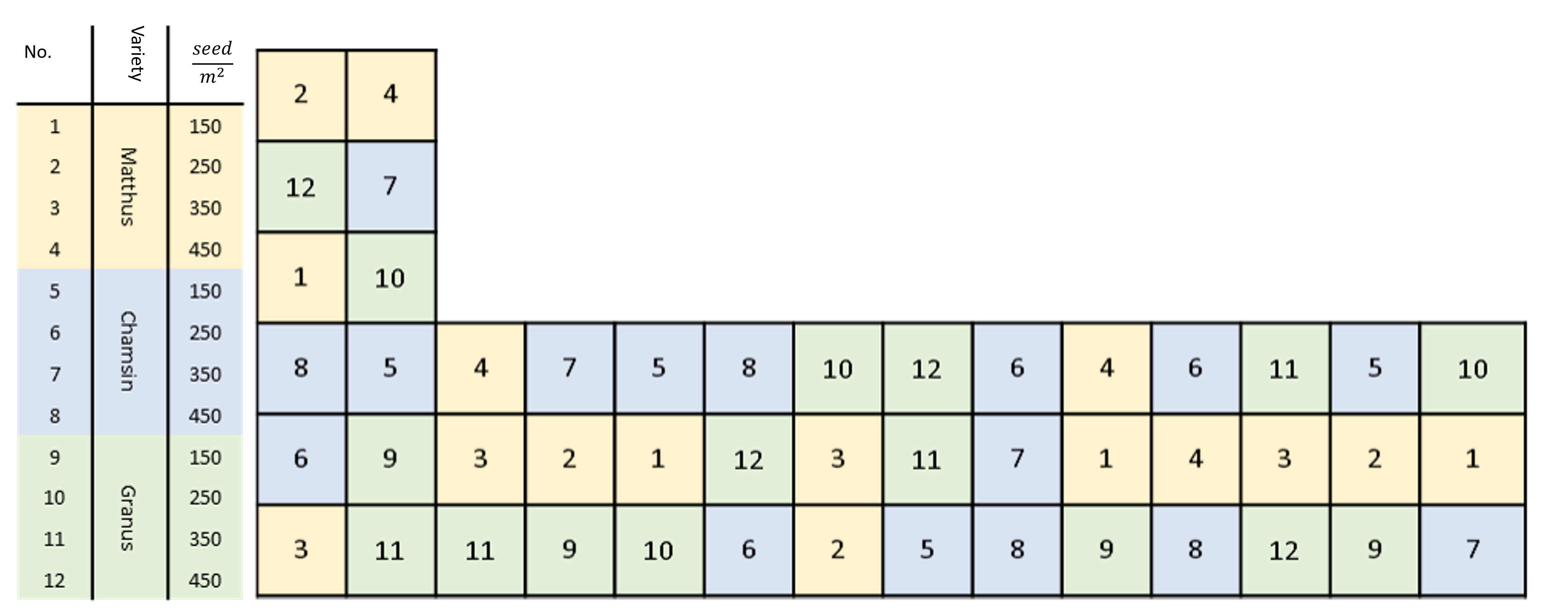

Supplement: Supplementary file 5 — Additional file 5. Color-coded plot design for field experiment. Three different varieties were sown with four sowing densities. The left Table shows the corresponding varieties and sowing densities. [file 13007_2023_1130_MOESM5_ESM.jpg]

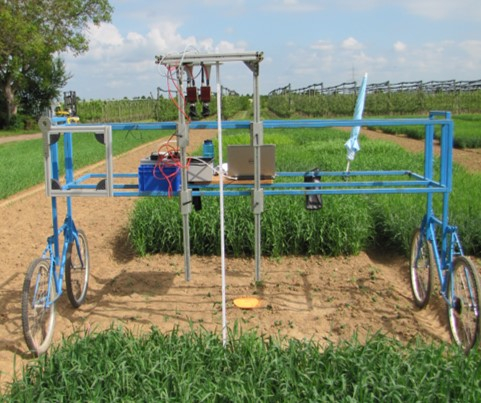

Supplement: Supplementary file 6 — Additional file 6. Stereo imaging setup mounted on the hand-driven “Field4Cycle”. [file 13007_2023_1130_MOESM6_ESM.jpg]

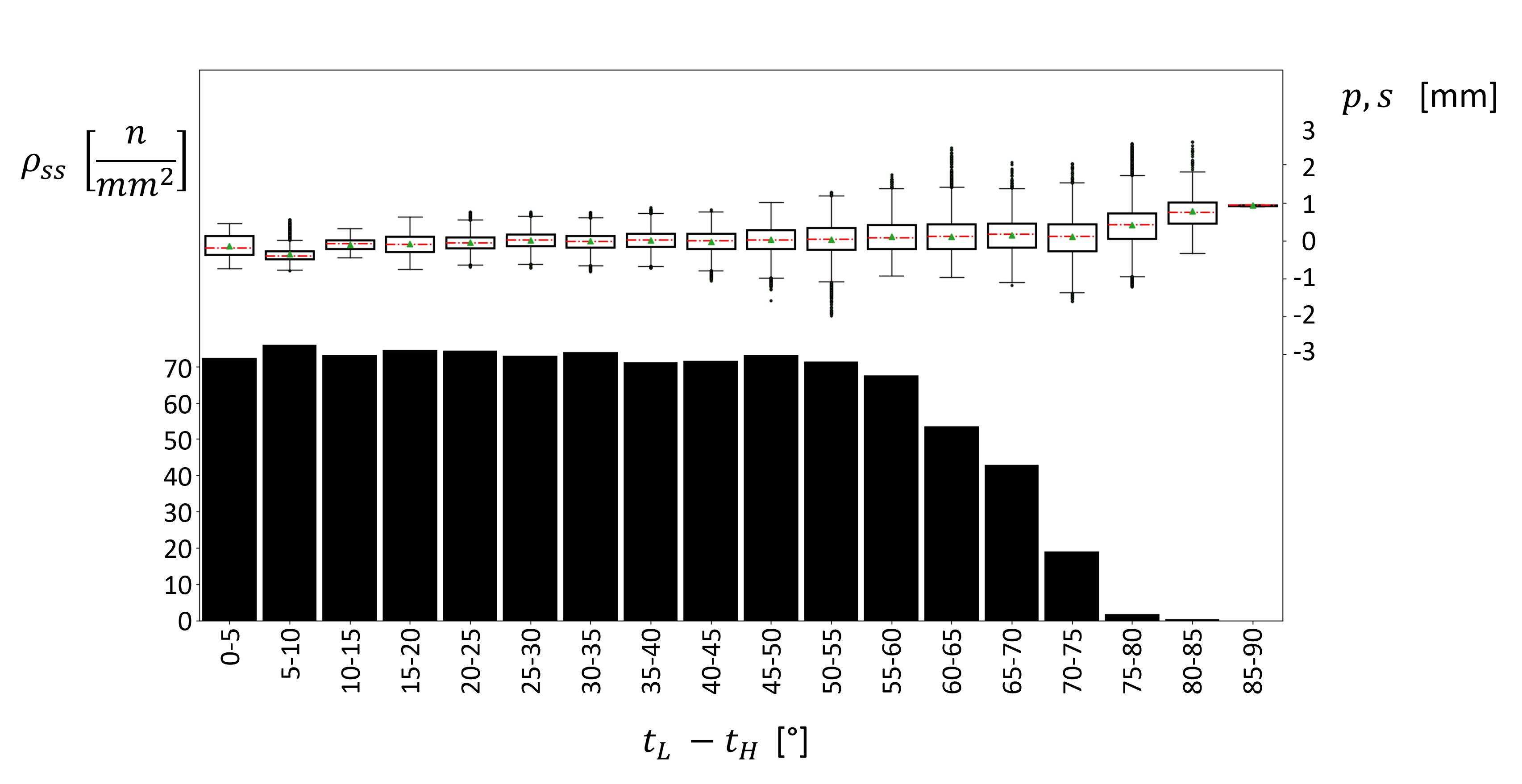

Supplement: Supplementary file 7 — Additional file 7. Point cloud density ${\rho }_{ss}$ρss for the projected area. The bar chart shows the point cloud density ${\rho }_{ss}$ρss for the projected (visual) area of different spherical segments. Segments were defined by ${i}_{r}$ir, segments ranged between ${t}_{L}$tL-${t}_{H}$tH. 3D points were reconstructed for all spherical segments. It is apparent that the number of points per projected area decreases for surfaces with an inclination angle above 60°. The boxplots in the upper part of the figure depicts the reconstruction error [file 13007_2023_1130_MOESM7_ESM.jpg]

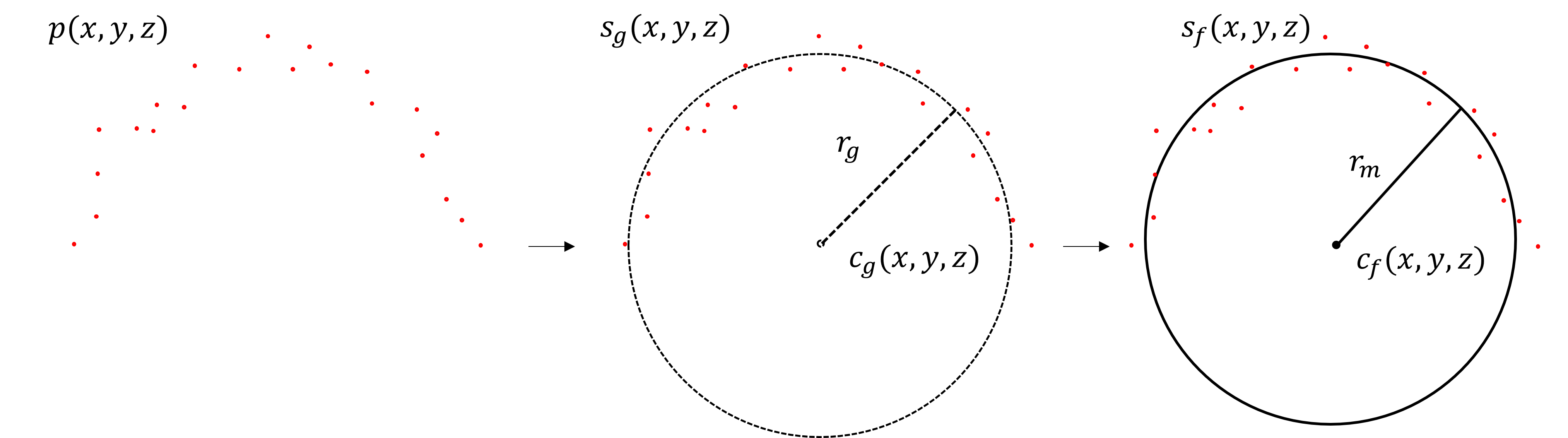

Supplement: Supplementary file 8 — Additional file 8. Process to approximate the real sphere center. A sphere ${s}_{g}$sg is fitted to the data points $p$p. The sphere is defined by ${c}_{g}$cg and ${r}_{g}$rg. Following this ${c}_{g}$cg is used as a starting point to fit a sphere ${s}_{f}$sf with the real radius ${r}_{m}$rm [file 13007_2023_1130_MOESM8_ESM.jpg]
