# Supplementary material for: Completing the picture of field-grown cereal crops: a new method for detailed leaf surface models in wheat
Source: Plant Methods. 2024 Feb 3;20:21. doi: 10.1186/s13007-023-01130-x (PMC10837940; doi:10.1186/s13007-023-01130-x)
Supplement: Supplementary file 9 — Additional file 9. Sphere reconstruction. [file 13007_2023_1130_MOESM9_ESM.docx]

# Supplementary

A sphere was used as validation target as it displays all inclination angles at its surface. For that reason, the sphere helped us to evaluate the processing pipeline with respect to the capability to reconstruct 3D point clouds from surfaces with the full range of inclination angles.

### Results

The accuracy of our approach was validated based on three criteria: i) the potential to reconstruct 3D points from surfaces with different inclination angles; ii) the point cloud density $\rho_{ss}$ for different surface inclinations, which is regarded as a prerequisite to reconstruct the respective surface; and iii) the mean error of the reconstructed inclination angle$d\left( p,s \right)$, which contains information on accuracy and precision of the reconstructed 3D point locations. The results are visualized for one representative measurement in Supplementary 7.

Based on the findings for $\rho_{ss}$ and $d\left( p,s \right)$, three intervals can be distinguished: In the first one with an inclination angle between 0° - 60°, $\rho_{ss}$ stays stable and the median of $d\left( p,s \right)$is < 1 mm. In the second interval from 60° - 75°, the median error of $d(p,s)$ stays stable, albeit $\rho_{ss}$ declines rapidly. For inclination angles > 75 ° $\rho_{ss}$ is almost zero and the median of $d(p,s)$ increases clearly. Based on these intervals, a range was defined, where $\rho_{ss}$ is sufficiently high for reliable surface reconstruction.

### Methods

A sphere was used in this experiment to determine the perspective limitations of the pipeline and address the slanted surface problem, which is well-known in stereo vision [34]. We assume that the reconstruction is less accurate for strongly inclined surfaces because the visual area relative to the real surface area decreases. To quantify this effect, the point cloud computed from the sphere target was analyzed segment-wise, meaning that the point cloud was split up into spherical segments each representing a particular inclination range of the sphere surface. Then, the visual area of this spherical segment was calculated, and the number of points were reconstructed from this surface area. This experiment provided information on the point cloud density per spherical segment. A sphere of 80.95 mm in diameter (measured with a *Vernier caliper set screw DIN 862, MIB Messzeuge Germany*) was imaged nine times with different positions within the field of view and different exposure times. Images were processed according to the described workflow resulting in the ground-level corrected point cloud $p$’. However, this step gave no information on the positional relationship between the sphere and its reconstructed counterpart which is required for the radius fitting process. Therefore, we approximated the coordinates of the real sphere surface by first estimating the sphere center position $c_{f}$ from the reconstruction and then combining it with the real sphere radius. First, a sphere $s_{g}$ was fitted to $p'$ via least squares regression. This was used as an initial guess for the sphere center position$c_{g}$. In the next step, the real sphere center $c_{f}$ was approximated by minimizing the mean distance between $p'$ and a sphere $s_{f}$, computed with $c_{g}$ and the true sphere radius $r_{m}=$40.475 mm, via least squares regression. We assumed that the corresponding sphere $s_{f}$represents the real sphere (Supplementary 8).

We computed a reconstruction error for each point, which is given by the Euclidian distance:

$$d\left( p,s \right)= \sqrt{{p_{x}-c_{fx}}^{2}+{p_{y}-c_{fy}}^{2}+{p_{z}-c_{fz}}^{2}}-r_{m}$$

between reconstructed points and *the* fitted surface. The sphere was cut into 18 spherical segment subsets $p_{ss}$ in steps of 5°. Lower and upper boundaries of the segments were identified by the inclination angle $i_{r}$ of the reconstructed data points. For the subsets, we calculated the inclination angle:

$$i_{r}=180-\arccos\left( \frac{p_{z}-c_{fz}}{r_{m}} \right)$$

for all 3D points and dedicated all points $p_{ss}$with $i_{r}$higher than $t_{L}$ and lower than $t_{H}$ to one subset

$p_{ss}=p\left( {t_{L}\leq i}_{r}\leq t_{H} \right) \left( 0\leq t_{L}\leq85 \right) (t_{H}=t_{L}+5)$.

For each spherical segment the point cloud density was determined via

$\rho_{ss}=\frac{O_{ss}}{n_{ss}}$,

where $O_{ss}$ is the visual area of each spherical segment from the surface area:

$O_{ss}=\pi*{r_{m}}^{2}*\left( {\sin\left( t_{H} \right)}^{2}-{\sin\left( t_{L} \right)}^{2} \right)$ with $\left( 0\leq t_{L}\leq85 \right), \left( t_{H}=t_{L}+5 \right)$

and $n_{ss}$ is the number of points in a given spherical segment.
